# Comprehensive characterisation of organic pollutants in high-polluted sediments and gull eggs using a broad-scope gas chromatography–Orbitrap methodology

**DOI:** 10.1007/s00216-026-06532-z

**Published:** 2026-05-09

**Authors:** Bernat Oró-Nolla, Francisco Javier Santos, Albert Bertolero, Sílvia Lacorte

**Affiliations:** 1 Department of Environmental Chemistry, IDAEA-CSIC, Jordi Girona 18-26, 08034 Barcelona, Catalonia Spain; 2 Department of Geosciences, University of Tübingen, Schnarrenbergstraße 94-96, 72076 Tübingen, Germany; 3 Department of Chemical Engineering and Analytical Chemistry, University of Barcelona, Martí i Franquès 1, Barcelona, Catalonia 08028 Spain; 4 Associació Ornitològica Picampall de Les Terres de l’Ebre, Trinquet 8, Deltebre, 43580 Spain

**Keywords:** High-resolution mass spectrometry, Sediments, Gull eggs, Targeted and non-targeted analysis

## Abstract

Polluted sediments affected by historic discharges of hexachlorobenzene (HCB), polychlorinated biphenyls (PCBs), dichlorodiphenyltrichloroethanes (DDTs), and polycyclic aromatic hydrocarbons (PAHs) pose risks to wildlife due to their bioaccumulative and toxic properties. Here, we propose an analytical method based on gas chromatography coupled to Orbitrap high-resolution mass spectrometry (GC–Orbitrap–HRMS) to investigate the presence of multiple contaminants in sediments from the Flix chlor-alkali plant hotspot and to assess their impact on birds from the Ebro Delta Natural Park using eggs of Audouin’s gull (*Larus audouinii*) as biomonitors. A combined target/suspect approach was optimised to (i) perform the quantitative analysis of 45 target persistent organic pollutants (POPs) and PAHs and evaluate quality parameters including recoveries, reproducibility, sensitivity, selectivity, and uncertainty measurement, and (ii) apply a suspect screening workflow using the high-resolution NIST library database to identify additional contaminants. Differences in compound patterns were observed between the matrices. In sediments, 4,4′-DDT was the predominant compound detected, reaching a maximum concentration of 49,386 ng g^−1^ dry weight (dw), while PCB congeners ranged from 0.06 to 1376 ng g^−1^ dw. In gull eggs, 4,4′-dichlorodiphenyldichloroethylene (4,4′-DDE) was detected from 1426 to 3473 ng g^−1^ dw, and PCBs levels ranged from 0.40 to 2190 ng g^−1^ dw. The suspect screening revealed 88 contaminants in the sediments (73 PCBs) and 43 compounds in the gull eggs (33 PCBs). Additional contaminants identified were plasticizers, industrial chemicals, and agrochemicals. This work demonstrates the importance of comprehensive contaminant characterisation in pollution hotspots to improve our understanding of potential impacts on wildlife inhabiting natural ecosystems.

**Graphical abstract:**

**Figure:**
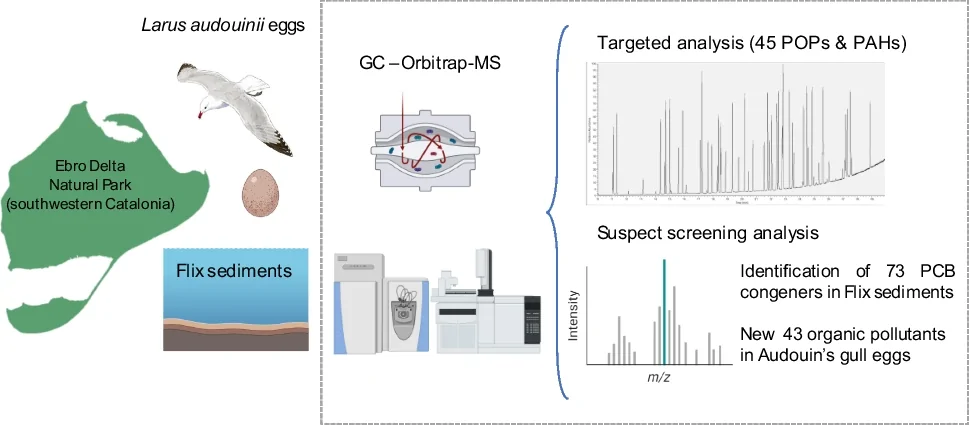

**Supplementary Information:**

The online version contains supplementary material available at https://doi.org/10.1007/s00216-026-06532-z.

## Introduction

The comprehensive characterisation of persistent organic pollutants (POPs) in environmental samples is essential for understanding their distribution, partitioning, and potential impact. POPs are complex organohalogenated compounds of different chemical families that comprise polychlorinated biphenyls (PCBs), organochlorinated pesticides (OCPs), solvents, and flame retardants, among others. Multiresidue analysis using gas chromatography (GC) coupled to mass spectrometry (MS) or tandem MS enables their identification. However, the advent of high-resolution mass spectrometry (HRMS) has significantly expanded the scope of compound characterisation capabilities through suspect screening analysis (SSA) and non-targeted analysis (NTA) [1–3]. These approaches rely on accurate mass measurements to improve the identification of known and emerging contaminants [4]. GC–Orbitrap mass spectrometry provides robust and reproducible measurements, operating at resolutions between 60,000 and 280,000 full-width at half maximum (FWHM, at *m/z* 200), and achieving mass accuracies below 5 ppm, and in some cases below 1 ppm [5–7]. The advantages include (a) expanded analyte coverage in a single analysis, (b) the capability to perform retrospective data analysis for the detection of analytes lacking reference standards, and (c) more streamlined data acquisition workflows [8]. Additionally, the use of extensive HRMS electron ionisation (EI) spectral libraries, such as the National Institute of Standards and Technology (NIST) database, enables the exact mass spectral matching and greatly expands the scope of compound identification [9, 10]. Chemical signatures can be confirmed at different confidence levels, as reported in the literature [11, 12].

Our study area is the Flix reservoir, located in the lower course of the Ebro River (southern Catalonia, Spain). This site is a well-known pollution hotspot that has affected the Ebro Delta, a major rice-growing field area and an important breeding area for more than 300 bird species [13, 14]. A chlor-alkali plant has been operating there since the early twentieth century and has historically discharged toxic wastes into the Flix reservoir, generating an environmental risk [15]. Between 1945 and 1971, approximately 35,000 tonnes of dichlorodiphenyltrichloroethane (DDT) were produced at the facility [16], and from 1959 to 1987, it was the only manufacturer of PCBs in Spain, producing a total of 29,000 tonnes of these compounds [17]. A dam constructed in 1948 led to the accumulation of industrial waste in the riverbed just directly in front of the plant [18]. The polluted sediments contained large amounts of OCPs and PCBs, as well as by-products from the synthesis of organochlorine compounds such as pentachlorobenzene (PeCB), hexachlorobenzene (HCB), polychloronaphthalenes, and polychlorostyrenes. They also contained heavy metals, including cadmium, arsenic, copper, chromium, selenium, lead (including radioactive ^210^Pb), and especially mercury [19]. In 2010–2011, a large-scale decontamination project was initiated, during which the riverbed sediments adjacent to the factory were confined within stainless-steel plates to create an artificial dam [20]. During the 2013–2015 period, an initial sediment remediation plan was implemented, involving the dredging and removal of around 1 million tons of polluted sediments across a 9-ha area [21]. A second phase took place between 2017 and 2019 to remove the remaining 100,000 tons of polluted material [22]. Analyses of sediments from the Flix dam conducted in 2006 reported high concentrations of major pollutants, including HCB (1900 ng g^−1^), PCBs (39,000 ng g^−1^), and all the 2,4′- and 4,4′-dichlorodiphenyldichloroethylenes (DDEs), dichlorodiphenyldichloroethanes (DDDs), and DDTs (1300 ng g^−1^) [19]. During the period 2021–2024, five plants equipped with vapour extraction systems were used to remove several tonnes of organochlorinated contaminants from sediments and groundwater [23]. Over the years, washing out processes and dredging activities remobilized POPs downstream along the Ebro River to its delta, 90 km away, with documented impact on various species, such as purple heron (*Ardea purpurea*) [24], Audouin’s and yellow-legged gulls (*Larus audouinii* and *Larus michahellis*) [25, 26], flamingos (*Phoenicopterus roseus*) [27], and catfish (*Silurus glanis*) [16, 28]. However, little is known about the wide-scope presence of multiple contaminants in these sediments and how their remobilization may have affected bird populations.

The specific aims are twofold: (1) to develop a combined target and suspect screening methodology to comprehensively identify and characterise multiple POPs and other contaminants in sediments from the Flix reservoir and (2) to determine their accumulation using eggs of the protected species Audouin’s gull (*Larus audouinii*) as bioindicators of environmental pollution. A method based on GC–EI–Orbitrap–HRMS was developed as follows: (i) calibration, assessment of the selectivity, sensitivity, and quantitative performance for the analysis of target POPs and polycyclic aromatic hydrocarbons (PAHs); (ii) evaluation of the solid–liquid extraction efficiency and method uncertainty using spiked sediments and hen eggs as quality control matrices (QCMs); and (iii) development of a suspect screening workflow to comprehensively increase the scope of analysis and identify additional pollution patterns in high-polluted sediments and gull eggs. The methodology proposed here has the potential capacity to detect hundreds of contaminants, providing essential information to better understand the impact of the Flix pollution hotspot downstream of the Ebro River, where rice fields and protected natural areas coexist.

## Materials and methods

### Chemicals and reagents

Target compounds analysed to set up the GC–EI–Orbitrap–HRMS method included 45 POPs, comprising 23 OCPs acquired from AccuStandard (New Haven, CT, USA), 8 PCB congeners acquired from Dr. Ehrenstorfer (Augsburg, Germany), and 14 PAHs acquired from Sigma-Aldrich (Darmstadt, Germany and St. Louis, MO, USA). Internal standards included ^13^C_6_–HCB, ^13^C_12_–4,4′–DDE, purchased from Cambridge Isotope Laboratories, Inc. (CIL, Andover, MA, USA), PCB 65 and PCB 209 acquired from Dr. Ehrenstorfer (Augsburg, Germany), and ^2^H_10_-acenaphthene, ^2^H_10_-phenanthrene, ^2^H_12_-chrysene, and ^2^H_12_-perylene obtained from Sigma-Aldrich (Darmstadt, Germany and St. Louis, MO, USA). The composition of the stock standards and working solutions of native and internal standards is indicated in Text S1 from the Supplementary Information (SI). *n*-Hexane (Hex), dichloromethane (DCM), and isooctane (ISO) for residue analysis were acquired from Merck (Darmstadt, Germany).

### Sample collection and preparation

**Fig. 1:**
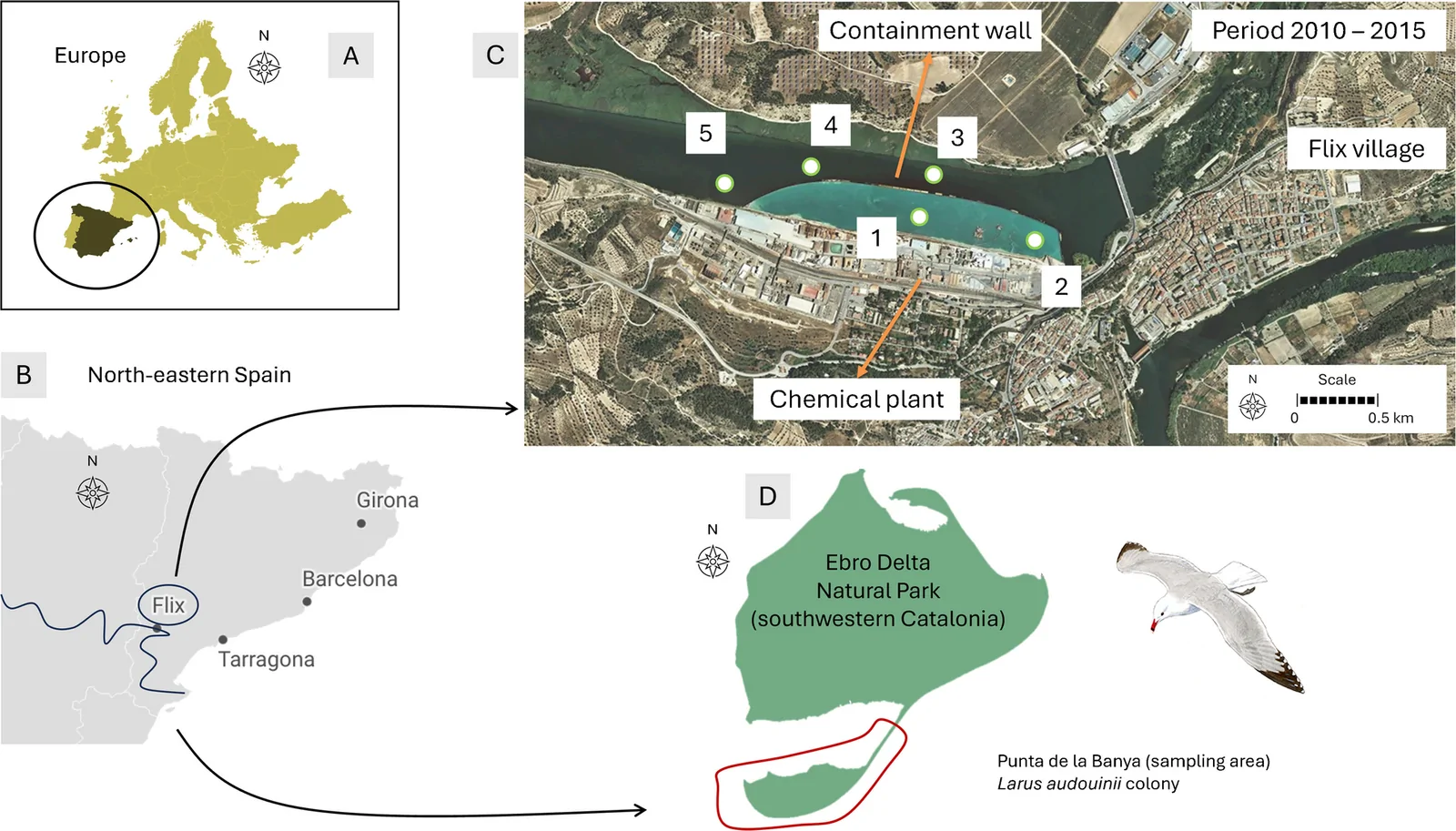
**A**) A map of Europe where Spain is located; **B**) a schematic location of Flix and the Ebro Delta Natural Park located in the north-eastern map of Spain; **C**) an aerial picture of the Flix dam is shown for the period 2010–2015, where sampling points are indicated; and **D**) the Ebro Delta Natural Park in red is indicated where the *Larus audouinii* colony breeds and from where the egg sampling is conducted

Figure 1 illustrates the location of the sampling areas, locating them in Europe (Fig. 1A) and in north-eastern Spain, in Catalonia (Fig. 1B). Samples analysed include five sediments collected in 2011 within the Flix reservoir (41° 13′ N, 00° 33′ E), after the containment wall was installed in front of the chlor-alkali plant. Figure 1C provides an aerial image showing the position of the artificial wall and the distribution of the sediment sampling points (Flix 1 and Flix 2 within the hydraulic barrier, and Flix 3, 4 and 5, outside the barrier with increasing distance upstream). Figure 1D shows the location of the Audouin’s gull breeding colony at Punta de la Banya, within the Ebro Delta Natural Park (southern Catalonia 40° 37′ N, 00° 35′ E). A total of 12 fresh eggs were collected at the beginning of the breeding season in 2012 and in 2019. For each year, two pooled samples of six eggs each were prepared and analysed.

Sample preparation consisted of weighing 0.5 g of freeze-dried sediment, sieved at 125 µm particle size, and 1 g of a freeze-dried egg. Each matrix was placed in a 50-mL glass centrifuge tube and fortified with 50 ng of the internal standard mixture. Extraction was performed with 30 mL of *n*-Hex:DCM (1:1, v/v) alternating 1 min of vortexing and 10 min in an ultrasonic bath, and this process was repeated three consecutive times [26]. These extracts were centrifuged at 3000 rpm for 10 min, and the resulting supernatant was collected and evaporated to 1 mL using a TurboVap® LV Concentration Evaporation Workstation (Biotage, Uppsala, Sweden) under a gentle N_2_ stream. Clean-up was carried out using Strata–Florisil cartridges (10 g, 60 mL; 170 µm particle size and 80 Å pore size) purchased from Phenomenex (Torrance, CA, USA), previously conditioned with 10 mL of *n-*hexane, and analytes were eluted with 30 mL of *n*-Hex:DCM (1:1, v/v). Purified extracts were concentrated to near dryness using the TurboVap®, employing 500 µL of isooctane as a keeper. The final extract was transferred to an amber 2-mL vial and stored at −21 °C until analysis. The water content of each gull sample was determined, and the mean value was 78.2%. Concentrations for sediments and eggs are expressed as ng g^−1^ dry weight (dw) for comparative purposes.

### GC–EI–full-scan HRMS analysis

Instrumental analysis was adapted from Oró-Nolla et al. (2024) [29]. Sediment and gull egg samples were analysed by GC–HRMS using a TRACE 130 GC coupled to a Hybrid Quadrupole-Orbitrap™ Mass Spectrometer (Thermo Fisher Scientific, Waltham, MA, USA). The chromatographic separation of the sample components was performed using a ZB-5MS fused silica capillary column (60 m length × 0.25 mm inner diameter × 0.25 µm film thickness) from Phenomenex (Torrance, CA, USA). A 60-m column was selected to allow a good chromatographic resolution and enable non-target screening, considering the large number of PCBs that could be expected in the analysed samples. The oven programme was set at 70 °C (hold for 3 min) and increased to 200 °C at 12 °C min^−1^ and to 315 °C at 6 °C min^−1^ (hold for 15 min). The analysis time was 45 min. The Orbitrap mass analyser was operated in EI mode at 70 eV. Data acquisition was performed in full-scan mode over the mass range *m/z* 70–1000 at a resolution of 60,000 (FWHM, at *m/z* 200). Instrument tuning and mass calibration were carried out using a perfluorotributylamine (PFTBA, CAS 311–89–7) solution, achieving a mass accuracy better than 0.5 ppm. The instrument control and data acquisition were performed using the Xcalibur™ version 4.1 (Thermo Fisher Scientific). Sequence programming and data processing were controlled using Tune 2.6 and Trace Finder version 4.1 software packages (Thermo Fisher Scientific). GC–EI–Orbitrap–HRMS spectra were compared to NIST 23 Mass Spectral Library & Search Software containing high-resolution EI mass spectra for about 347,100 unique compounds [10].

### Quality control analysis and validation

For quantification, a set of five calibration solutions containing all the target compounds (OCPs, PCBs, and PAHs) at concentrations ranging from 20 to 500 ng mL^−1^ and the internal standards at 100 ng mL^−1^ was prepared in isooctane by dilution of the corresponding standard mixtures. Table S1 indicates the standards used for the internal standard quantification. Instrumental repeatability and reproducibility were evaluated by consecutively injecting a 40 ng mL^−1^ standard mixture solution on the same day (*n* = 5) and on 3 days (*n* = 3 days × 5 replicates), respectively. Instrument detection limits (IDLs) were estimated using a signal/noise ratio (S/N ratio) of 3. Quality control and assurance were evaluated by analysing sediments from a pristine river in the Pyrenees (north Catalonia, Spain) and freeze-dried hen eggs, both used as quality control material (QCM). These two matrices spiked at 50 ng g^−1^ dw for sediments and 40 ng g^−1^ dw for eggs (*n* = 5), respectively, were used for method validation. Method detection limits (MDLs) were calculated using a signal/noise (S/N) ratio of 3 based on the analysis of the two QCM samples. Procedural blanks covering both instrumental and sample preparation procedures were analysed in each extraction batch to evaluate the contribution of background levels on the response. In addition, sample blanks (one clean sediment from a mountain river and one hen egg) were analysed to assess possible interferences from matrix components. All blanks were spiked with 50 ng of internal standards and analysed using the proposed method.

The uncertainty of the method for POPs and PAHs was calculated by analysing QCMs under repeatability conditions (*n* = 5). The European Union SANTE 11312/2021 guideline for the estimation of measurement uncertainty of results was used as a reference [30]. The expanded uncertainty $\left(U^{\prime }\right)$ was estimated using a coverage factor of *k* = 2 and includes the measurements of the uncertainty components for the bias $$({u'}_b)$$ and for the precision $$({u'}_s)$$. Details of the measurements and calculations associated with these parameters have been reported previously [29].

### Identification criteria and suspect screening approach

Identification of the target compounds and additional potential chemicals was carried out according to the following criteria: (i) identification of the molecular ion or the base peak (specific for each compound) in the full-scan spectra with a mass tolerance of 5 ppm, an abundance > 10^6^ arbitrary unit (AU), and a minimum of 12 points/peak (FWHM); (ii) retention time check, allowing a 20% variation from the standard; (iii) detection of the other two most abundant and selective ions, established as the qualifier ions; (iv) complying with the isotope ion ratio (difference < 10%); (v) matching full-scan mass spectra to the NIST reference spectral library. The mass measurement errors (*Δm*_*i*_) were calculated according to Oró-Nolla et al. (2024) [29].

For the suspect screening approach, *Deconvolution Plugin* from the Trace Finder software was employed [31, 32]. Additionally, data were filtered through the following criteria:

1. to resolve all the *m/z* traces to obtain pure mass spectra with a 5 ppm tolerance in the mass extraction window and an ion abundance > 10^6^ AU as isolation conditions for peak deconvolution
2. to identify unique compounds according to a total score combining (a) the reversed search index (RSI) > 750, which specifies the results of a library comparing the mass spectrum of an unknown compound to an exact mass spectral library (i.e. NIST), and (b) the reversed high-resolution filtering (RHRF) score > 90, which represents the difference between the accurate mass (measured mass) and the exact mass (theoretical mass) in accordance with the elemental composition of the specific molecule
3. to select the mass traces with the molecular ion or base peak at a mass error of 5 ppm

Identified compounds were classified according to confidence levels [11]. To prove the robustness of the suspect screening methodology and to validate the procedure, a standard solution at 40 ng mL^−1^ and the QCM extracts for sediments and eggs were used to verify the identification and compound assignment.

## Results and discussion

### Analytical performance for the target approach

GC–EI–Orbitrap–HRMS performs at a very high sensitivity and selectivity allowing the unequivocal identification of pollutants at low concentration [33]. Table 1 summarises the quality parameters obtained for the overall analytical method used to determine the target compounds in sediment and egg samples. Good linearity was achieved in the 20–500 ng mL^−1^ range with a determination coefficient (*R*^2^) higher than 0.999 for all the compounds. Repeatability and reproducibility values expressed as relative standard deviation (RSD%) were all < 20%. IDLs ranged from 0.001 to 0.257 ng mL^−1^. Contributions of the analytes to the blanks were lower than the IDLs and thus, no blank subtractions were required. Figure S1 shows the GC-HRMS chromatogram of the total ion current (TIC) for a standard solution at 500 ng mL^−1^ with good chromatographic separation with the 60-m column and resolution for all the target compounds. Identification criteria for full-scan GC–EI–Orbitrap–HRMS analysis were achieved considering the retention time and the three most intense exact mass ions for each analyte. The base peak was used for quantification, and the other two most abundant and selective ions were established as the qualifier ions. In this study, the Δ*m*_*i*_ was < 1.84 ppm for quantification and confirmation ions for all the compounds (Table S2). Method detection limits for sediments (MDL_sed_) were between 0.01 and 0.33 ng g^−1^ dw, while for egg samples, MDL_eggs_ were from 0.05 to 2.39 ng g^−1^ dw. The proposed extraction method provided efficient recoveries (69–121%) for most of the target compounds (38 in sediments and 39 in hen eggs out of 45 analysed) with RSD% values < 18%. For Dieldrin, the main ion at *m/z* 79 was first selected as the quantification ion because it is the most intense fragment, but its intensity was low, presumably influenced by a low trapping efficiency of low molecular ions in the C-trap, which may lead to false identification [34]. Consequently, the ion at *m/z* 263 was selected for quantification, but the recoveries obtained were very low (13% in sediments and 33% in egg samples).

**Table 1:** Compounds studied, retention time, slope of the calibration curve, repeatability, reproducibility, instrumental detection limit (IDL), recoveries (relative standard deviation, %), method detection limit (MDL), and expanded uncertainty (*U′*) for sediments and eggs, respectively

Compounds	Retention time (min)	Instrumental parameters				Sediments			Eggs		
		Slope	Repeatability (*n* = 5, RSD%)	Reproducibility (*n* = 3, RSD%)	IDL (ng mL^−1^)	Recovery (*n* = 5, RSD%)	MDL (ng g^−1^ dw)	*U′* %	Recovery (*n* = 5, RSD%)	MDL (ng g^−1^ dw)	*U′* %
HCBu	11.34	0.398	0.9	3.0	0.107	87 (18)	0.01	60	114 (9.2)	0.32	41
Acenaphthylene	14.35	0.789	0.4	4.6	0.003	98 (6.8)	0.01	21	104 (3.9)	0.05	15
Acenaphthene	14.72	0.581	0.5	3.6	0.003	103 (1.2)	0.01	7	102 (3.4)	0.05	11
PeCB	15.00	0.681	0.5	1.4	0.002	109 (9.2)	0.02	34	117 (2.3)	0.14	34
Fluorene	15.89	0.667	0.7	6.8	0.004	111 (5.2)	0.01	27	118 (2.2)	0.05	36
α-HCH	17.13	0.346	0.4	2.0	0.006	99 (4.9)	0.05	15	95 (4.6)	0.23	17
HCB	17.24	0.685	0.5	0.6	0.005	115 (5.3)	0.07	34	115 (9.8)	0.09	44
β-HCH	17.66	0.265	0.8	2.3	0.008	95 (5.9)	0.06	21	98 (6.3)	0.32	19
γ-HCH	17.92	0.301	0.8	1.8	0.007	99 (4.3)	0.05	14	102 (3.7)	0.28	12
Phenanthrene	18.37	0.604	0.4	3.2	0.003	101 (4.3)	0.01	13	116 (7.0)	0.05	39
Anthracene	18.52	0.563	1.1	3.9	0.004	100 (0.6)	0.01	2	115 (1.6)	0.05	31
δ-HCH	18.55	0.270	0.6	1.0	0.009	99 (5.6)	0.06	17	95 (4.6)	0.37	17
PCB28	19.35	0.967	0.7	1.3	0.005	114 (1.6)	0.02	30	114 (1.1)	0.18	30
Heptachlor	19.78	0.168	1.6	13	0.061	120 (1.5)	0.20	40	111 (1.6)	0.73	22
PCB52	20.20	0.675	0.6	0.4	0.005	107 (1.4)	0.04	16	106 (1.3)	0.14	14
Aldrin	20.76	0.278	0.6	8.9	0.057	69 (5.9)	0.06	64	112 (0.2)	0.23	24
Isodrin	21.56	0.262	0.7	14	0.045	43 (12)	0.06	> 100	100 (3.9)	0.50	12
Heptachlorepoxide	21.77	0.224	0.5	8.2	0.159	49 (5.8)	0.13	> 100	62 (11)	0.87	> 100
Oxychlordane	21.88	0.059	0.8	2.2	0.257	99 (1.7)	0.33	5	112 (3.0)	2.39	27
Fluoranthene	22.06	0.721	1.3	2.6	0.003	93 (3.8)	0.01	18	114 (2.0)	0.05	29
2,4′-DDE	22.38	1.079	0.5	1.6	0.012	111 (1.0)	0.02	21	112 (0.5)	0.14	23
α-Chlordane	22.41	0.381	0.5	1.0	0.120	103 (0.9)	0.15	7	108 (1.2)	0.60	16
PCB101	22.50	0.735	0.2	1.2	0.049	102 (2.4)	0.05	10	98 (1.7)	0.18	5
β-Chlordane	22.77	0.300	0.8	0.6	0.160	106 (0.8)	0.20	13	111 (0.9)	0.83	22
Pyrene	22.82	0.753	1.0	3.8	0.002	86 (3.6)	0.01	29	113 (4.4)	0.05	29
4,4′-DDE	23.27	0.892	0.1	2.1	0.014	110 (1.3)	0.03	21	111 (2.9)	0.18	24
2,4′-DDD	23.48	0.682	0.8	9.3	0.018	121 (1.6)	0.06	43	127 (2.2)	0.28	54
Dieldrin	23.57	0.116	3.4	0.8	0.116	13 (2.1)	0.28	> 100	33 (18)	1.61	> 100
PCB118	24.25	0.917	1.0	3.4	0.042	108 (1.6)	0.04	17	106 (0.8)	0.18	12
4,4′-DDD	24.47	0.345	1.4	9.5	0.037	126 (1.0)	0.12	51	127 (2.7)	0.64	54
2,4′-DDT	24.57	0.712	1.1	6.8	0.019	116 (3.6)	0.05	33	111 (2.6)	0.28	24
PCB153	24.83	0.733	0.7	7.7	0.110	101 (2.3)	0.03	8	101 (1.9)	0.37	7
4,4′-DDT	25.56	0.254	2.4	15	0.052	116 (15)	0.15	57	103 (14)	0.92	43
PCB138	25.61	0.661	1.3	7.2	0.102	97 (0.9)	0.04	4	97 (1.7)	0.37	6
Methoxychlor	27.11	0.385	2.0	14	0.022	28 (9.3)	0.10	> 100	38 (16)	0.92	> 100
1,2-Benzanthracene	27.17	0.721	0.0	2.5	0.002	121 (2.7)	0.01	43	109 (1.5)	0.05	19
Chrysene	27.30	0.688	1.0	4.3	0.001	84 (1.1)	0.01	32	97 (0.9)	0.05	6
PCB180	27.50	0.521	0.7	7.0	0.103	105 (1.1)	0.12	12	106 (1.8)	0.37	14
Mirex	28.86	0.635	2.0	7.5	0.019	99 (3.2)	0.07	10	101 (3.4)	0.28	10
Benzo[b]fluoranthene	31.01	0.685	5.5	5.8	0.002	91 (3.5)	0.01	22	98 (2.2)	0.05	8
Benzo[k]fluoranthene	31.11	0.667	6.6	4.0	0.002	120 (2.7)	0.01	41	110 (2.0)	0.05	21
Benzo[a]pyrene	32.09	0.529	4.3	4.2	0.006	33 (7.4)	0.01	> 100	62 (15)	0.05	> 100
Indeno[1,2,3-cd]pyrene	35.82	0.320	3.6	16	0.005	93 (5.9)	0.01	22	117 (7.1)	0.09	42
Dibenz[a,h]anthracene	35.94	0.289	1.9	16	0.094	109 (14)	0.03	48	101 (13)	0.23	38
Benzo[g,h,i]perylene	36.78	0.428	3.6	5.0	0.003	9 (14)	0.02	> 100	82 (12)	0.05	48

Regarding method uncertainty, most pollutants (35 in sediments and 39 in eggs out of 45 compounds analysed) showed $U{\prime}$ values ≤ 50%, indicating, as recommended by SANTE 11312/2021 [30], a good performance of the proposed analytical method for both matrices (Table 1). As commercial analytical standards were used to spike the samples, it was assumed that the uncertainty associated with the spiking levels was negligible. $U{\prime}$ values > 100% were obtained for heptachlor epoxide and methoxychlor due to low recoveries (28 to 62%). In these cases, the uncertainty is strongly influenced by the deviation from the spiked value (bias) rather than by the precision among measured replicates. Table S3 shows that components for bias $({u'}_b)$ ranged from 0.8 to 31% for 39 compounds in sediments and from 1.9 to 38% for 43 compounds in eggs, indicating that recovery values were generally close to the theoretical spiked concentration, except for isodrin, heptachlorepoxide, dieldrin, and methoxychlor (in both matrices), as well as benzo[a]pyrene and benzo[g,h,i]perylene, for which the bias ${u'}_b$ was > 50%. The precision component ${u'}_s$ accounts for the variability of measurements using the spiked sediments and eggs to calculate precision under methodological repeatability conditions, and there is no real or true value associated with this variable. If the ${u'}_s$ value is low, the performance is good since replicates have less dispersion. In this study, the component ${u'}_s$ ranged from 0.7 to 22% for sediments and from 0.2 to 28% for eggs, for all the compounds. The fact that the bias component was more variable than the precision component is related to the good reproducibility obtained (RSD < 20%), while the bias is related to the proximity to the spiked value, and compounds not efficiently recovered cause an increase in the ${U'}$ values. Consequently, the combined uncertainty component ${u'}$ is mostly affected by bias than by precision.

Overall, the calculation of $U{\prime}$ provides an additional quality parameter demonstrating the suitability of the proposed methodology for the determination of POPs and PAHs in sediments and egg samples, enabling analytical validation and comparability of results. Previous studies in blood reported values of $U{\prime}$ from 21 to 47% for POPs and PAHs using GC–Orbitrap [29], and in fish, $U{\prime}$ ranged from 14 to 29% for OCPs and polybrominated biphenyl ethers (PBDEs) using GC–MS/MS [35], and from 4.8 to 15% for HCB, HCBu, PeCB, and HCHs using GC–Orbitrap [28, 35]. Overall, the GC–EI–Orbitrap–HRMS method provided high confidence in the identification of POPs and PAHs, even though eggs and sediments are complex matrices.

### Concentration of sediments and gull eggs from the Ebro Delta

**Fig. 2:**
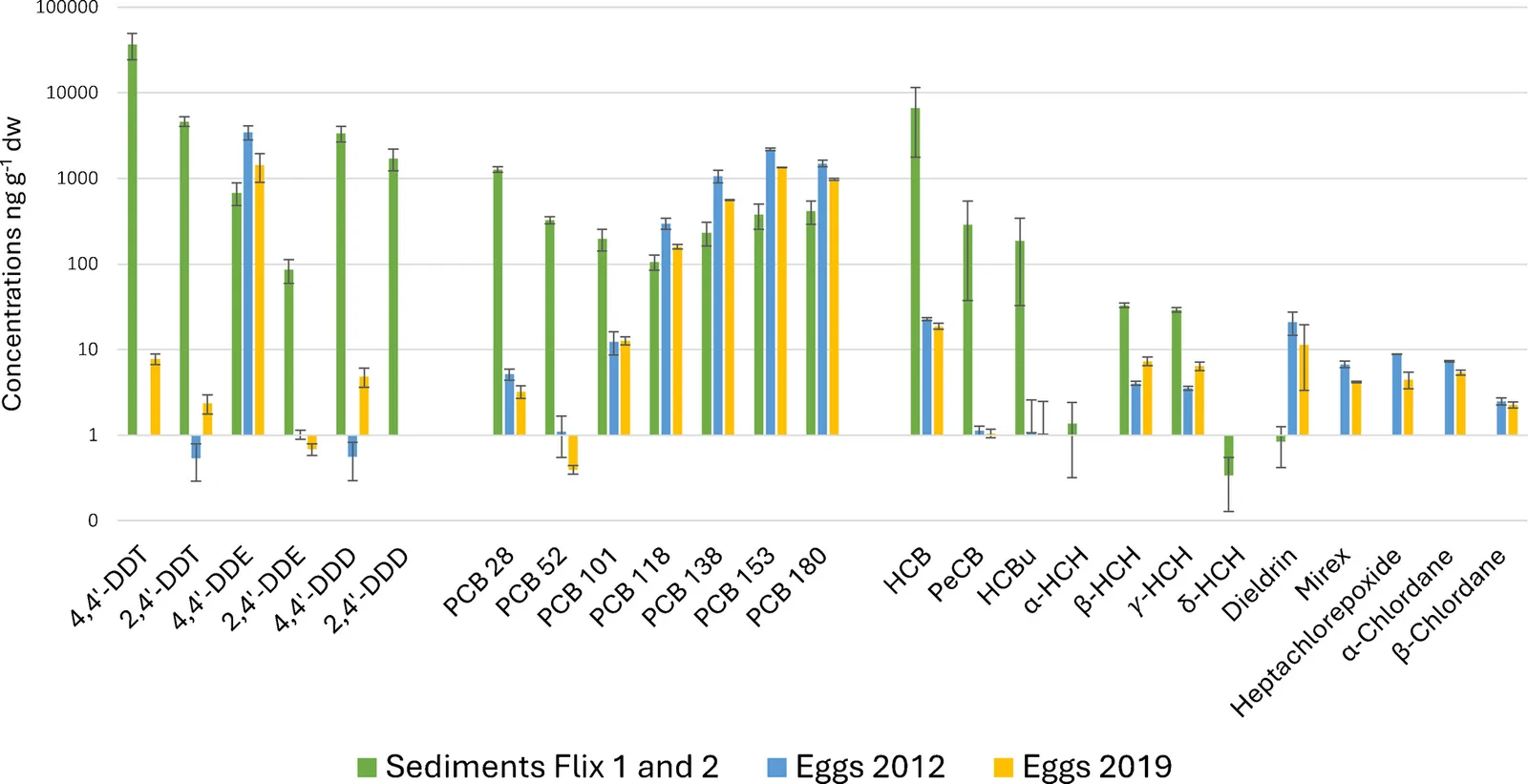
Comparison of the mean concentrations (ng g^−1^ dw, log_10_ scale) of target contaminants in sediments Flix 1 and 2 and the concentrations in gull eggs from 2012 and 2019, with included standard deviation from the two average values, respectively, for the two sediments and the two eggs, for each year

Figure 2 compares the mean concentration of target contaminants in sediment Flix 1 and 2 and the concentration in gull eggs from 2012 and 2019. It can be seen that the profile is very different in both matrices, where sediments accumulate all DDTs, PCBs, and solvents produced in the plant, while eggs accumulate 4,4′-DDE and other OCPs not necessarily originating from the plant, and the most accumulative PCBs. While contamination patterns are distinct in the two matrices, it can be noted that sediments are a primary pollution source of POPs, underscoring both the persistence and availability of these pollutants and the likelihood of ongoing trophic transfer within the Ebro Delta ecosystem. Also, it is observed that remediation activities have changed the concentrations of POPs in gull eggs but not the patterns, indicating a long-term exposure with pervasive high concentrations in gull eggs.

Table 2 shows the concentration of pollutants in the five analysed sediments from Flix reservoir and in gull eggs, and only compounds detected in both or one or another matrix are included. In sediments, the ∑POPs concentrations ranged from 14.7 to 62,132 ng g^−1^ dw. The most polluted sample was Flix 2, followed by Flix 1 (both within the hydraulic barrier) while the remaining sites showed lower concentrations in the order Flix 3 > Flix 4 > Flix 5. Clearly, the ∑POPs concentration was orders of magnitude higher in the confined sediments (Flix 1 and 2) than in the sediments outside the barriers (Flix 3 to 5), and this indicates that the limits of the barrier included the area with the polluted residues. The most ubiquitous compounds were 4,4′-DDT, PCBs, HCB, hexachlorobutadiene (HCBu), PeCB, and hexachlorocyclohexanes (HCHs). Despite the production of DDTs being finalised in 1971 [16], for these 2011 samples, concentration ranges were 1.65–49,386 ng g^−1^ dw for DDTs, 0.06–882 ng g^−1^ dw for DDEs, and 0.23–4,073 ng g^−1^ dw for DDDs, and decreasing concentrations were observed according to increasing distance from the plant (as shown in Fig. 1C), where for Flix 5, some DDTs were not detected. Flix 2 had the highest 4,4′-DDT concentration, and this might account for proximity to the release pipe from the plant or the direction of the river flow. Among the PCBs, the profile was dominated by PCB28 with 0.28–1376 ng g^−1^ dw followed by PCB180 > PCB153 > PCB52 > PCB138 > PCB101 > PCB118 with concentrations from 0.06 to 540 ng g^−1^ dw and again the concentration decreased 1000 times from Flix 1 to 5. High-chlorinated PCBs present in the sediment suggest that these chemicals were transported through the Ebro River from Flix to its delta, affecting Audouin’s gull but potentially also other species in the area.

**Table 2:** Concentrations of detected contaminants in Flix sediments (*n* = 5) expressed as ng g^−1^ dw, and in gull eggs (*n* = 4) indicated as ng g^−1^ dw, average concentrations and standard deviation values, respectively. *n.d.* indicates not detected

Compound	Sediments					Eggs 2012	Eggs 2019
	Flix 1 ng g^−1^ dw	Flix 2 ng g^−1^ dw	Flix 3 ng g^−1^ dw	Flix 4 ng g^−1^ dw	Flix 5 ng g^−1^ dw	Mean concentration (standard deviation) ng g^−1^ dw	Mean concentration (standard deviation) ng g^−1^ dw
HCBu	342	32.7	30.4	4.27	1.09	1.07 (1.51)	1.02 (1.45)
PeCB	543	37.3	7.76	0.22	0.18	1.14 (0.14)	1.05 (0.12)
α-HCH	2.41	0.32	n.d.	n.d.	n.d.	n.d.	n.d.
HCB	11,512	1,762	35.6	3.23	3.55	22.7 (0.87)	18.7 (1.54)
β-HCH	35.1	31.0	0.37	0.09	n.d.	4.03 (0.22)	7.31 (0.84)
γ-HCH	30.9	27.3	0.33	0.08	n.d.	3.55 (0.20)	6.43 (0.74)
δ-HCH	0.13	0.55	n.d.	n.d.	n.d.	n.d.	n.d.
PCB28	1376	1171	16.7	0.46	0.28	5.17 (0.77)	3.24 (0.55)
PCB52	355	294	10.6	n.d.	0.28	1.11 (0.56)	0.40 (0.05)
Heptachlorepoxide	n.d.	n.d.	n.d.	n.d.	n.d.	8.80 (0.08)	4.48 (0.98)
2,4′-DDE	112	59.8	7.1	0.06	n.d.	1.02 (0.13)	0.68 (0.11)
α-Chlordane	n.d.	n.d.	n.d.	n.d.	n.d.	7.27 (0.16)	5.38 (0.37)
PCB101	253	142	18.4	n.d.	n.d.	12.4 (3.72)	12.7 (1.42)
β-Chlordane	n.d.	n.d.	n.d.	n.d.	n.d.	2.50 (0.23)	2.26 (0.19)
4,4′-DDE	882	479	45.6	1.99	2.03	3473 (656)	1426 (533)
2,4′-DDD	2201	1232	205	1.17	0.23	n.d	n.d
Dieldrin	1.26	0.42	n.d.	n.d.	n.d.	21.1 (6.30)	11.5 (8.11)
PCB118	127	84.7	9.29	0.16	0.06	299 (43.9)	159 (9.33)
4,4′-DDD	4073	2645	589	0.37	3.41	0.54 (0.25)	2.35 (0.59)
2,4′-DDT	5222	4041	783	9.84	1.65	0.56 (0.27)	4.86 (1.22)
PCB153	504	254	41.2	0.57	0.89	2190 (63.8)	1354 (0.79)
4,4′-DDT	24,341	49,386	13,967	5.09	n.d.	n.d.	7.80 (1.10)
PCB138	305	162	26.0	0.35	0.42	1063 (178)	557 (5.48)
PCB180	540	289	51.9	0.68	0.67	1503 (136)	973 (27.6)
Mirex	0.14	n.d.	n.d.	n.d.	n.d.	6.72 (0.60)	4.18 (0.07)
∑POPs	52,758	62,132	15,845	28.6	14.7	8628	4563

In the case of HCB, it was detected from 11,512 (Flix 1) and decreased to 3.23 and 3.55 ng g^−1^ dw with distance to the plant (Flix 4 and 5), and the same behaviour was observed for HCBu and PeCB that decreased ten times from Flix 1 (342 and 543 ng g^−1^ dw) to Flix 2 and 100 times outside the containment wall with relatively low concentrations (1.09 and 0.18 ng g^−1^ dw). α-, β-, γ-, and δ-HCH isomers were found at much lower concentrations compared to other OCPs, with higher concentrations in sediments inside the wall (up to 35.1 ng g^−1^ dw) than outside, and β-HCH and γ-HCH were the most prevalent isomers. Mirex was only detected in Flix 1 at concentrations close to the MDL, and heptachlor epoxide and chlordanes were not detected in sediments and only in gull eggs. PAHs were not detected in the Flix sediments.

**Fig. 3:**
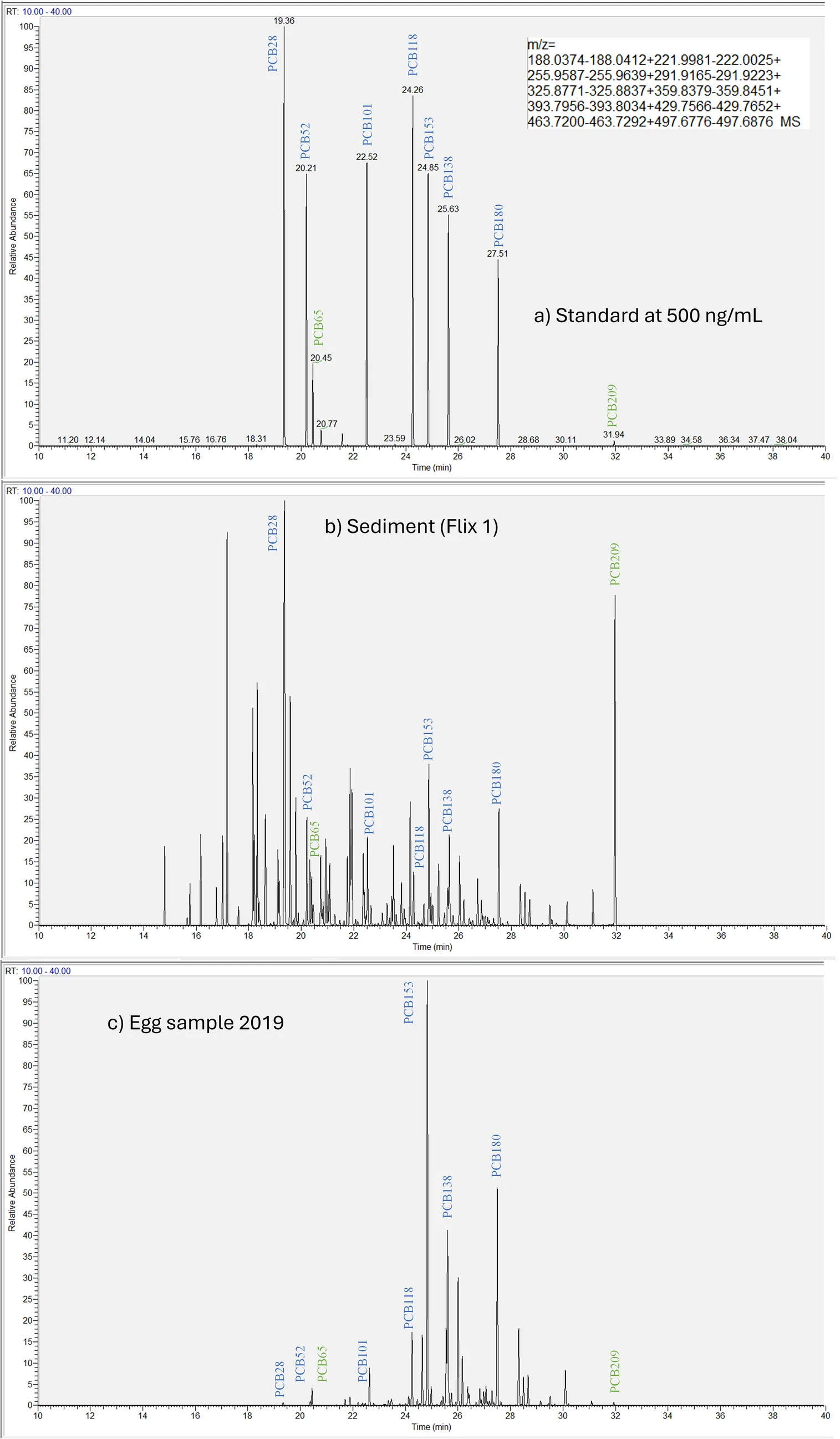
GC–EI–Orbitrap–MS extracted ion chromatogram for PCBs (molecular ions selected) of each chlorination level, for **a** a standard at 500 ng/mL, **b** a sediment sample (Flix 1), and **c** a 2019 egg sample

Regarding the concentrations of the target compounds in eggs collected in 2012 and 2019 (Table 2), the ∑POPs in the 2012 gull eggs were approximately twice those measured in 2019 samples. PCBs were the most prevalent compounds in both years, with the congener profile dominated by PCB153 > PBC180 > PCB138. Concentrations ranged from 1063 to 2190 ng g^−1^ dw in 2012 and decreased to 557–1,354 ng g^−1^ dw in 2019. This congener pattern is consistent with the higher bioaccumulation potential of hexa- and heptachlorobiphenyls compared with less chlorinated congeners. Specifically, PCB118 ranged from 159 to 299 ng g^−1^ dw, while PCB28, PCB52, and PCB101 were found below 15 ng g^−1^ dw, considering both years. Figure 3 shows differences in the PCBs’ chromatographic profiles, comparing with a standard mixture, where the sediment displays a higher abundance and diversity of PCB features, whereas the gull egg is mainly dominated by marker congeners. This example evidences the differential profiles in sediment and gull eggs.

Regarding the OCPs, 4,4′-DDE was detected at 3473 ng g^−1^ dw in 2012 and decreased to 1426 ng g^−1^ dw in 2019. 4,4′-DDT was only detected in 2019 at 7.80 ng g^−1^ dw, confirming that this highly persistent compound can still be found in its precursor form more than 50 years after being produced. In both years, 4,4′-DDE concentrations were much lower than 4,4′-DDT that was prevalent in the sediments, indicating metabolization in gulls, which is consistent with previous studies [24–26]. Other compounds, such as 4,4′-DDD, 2,4′-DDE, and 2,4′-DDT, were detected in gull eggs at concentrations lower than 10 ng g^−1^ dw, whereas 2,4′-DDD was not detected. A similar pattern was also noted for β-HCH and γ-HCH.

HCB was detected at 22.7 ng g^−1^ dw in 2012 and 18.7 ng g^−1^ dw in 2019, suggesting low-level exposure over the years. Despite HCB, PeCB, and HCBu showing high concentrations in sediments, their concentrations in eggs were much lower, likely due to low bioaccumulation potential and high volatility [19]. Dieldrin, one of the most used pesticides in the past, was found at maximum concentrations of 21.1 and 11.5 ng g^−1^ dw in 2012 and 2019, respectively. Although Dieldrin was never produced in Flix, it was more prevalent in eggs than in sediments, likely reflecting past agricultural use in the surrounding rice fields. Its presence is of concern, as Dieldrin poses particular risks to high-trophic-level birds [36]. Heptachlor epoxide, chlordanes, and mirex were detected at < 10 ng g^−1^dw exclusively in egg samples, potentially originating from agricultural activities in this area rather than from contaminated sediments where they were not detected. No PAHs were detected in *Larus audouinii* eggs.

A temporal comparison of gull egg concentrations between 2012 and 2019 reveals a 50% concentration decrease in legacy POPs levels, particularly for 4,4′-DDE and highly chlorinated PCB congeners. This means that the remediation activities have been effective in diminishing the POPs burden in gulls. As gull eggs serve as bioindicators of POPs pollution in the bird breeding area of the Ebro Delta Natural Park, the impact of these results is huge for ensuring the well-being of the ecosystem. These findings further confirm that gull species function as effective biomonitors of organic contamination in the region.

### Evaluation of the suspect screening methodology

**Fig. 4:**
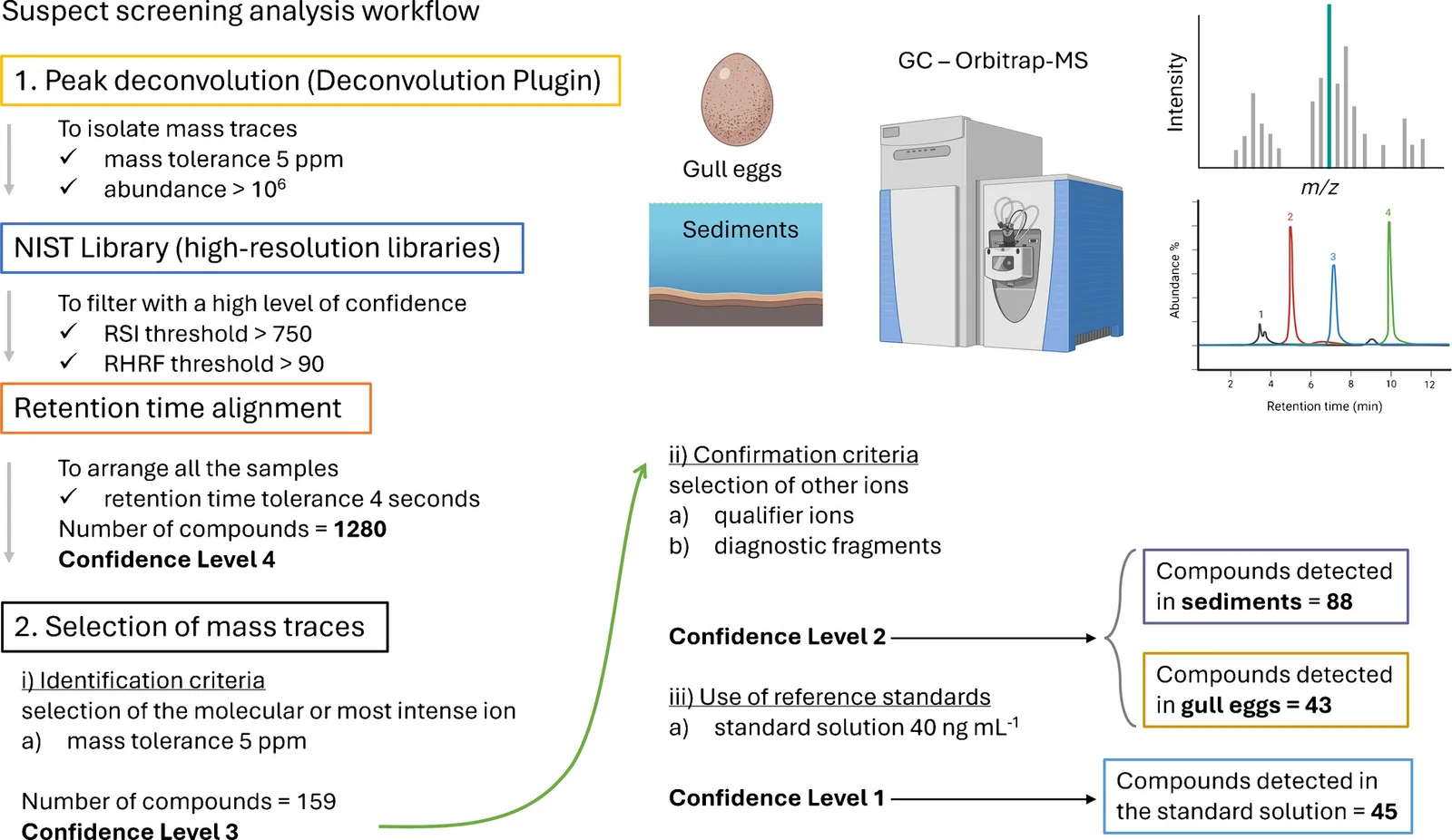
Workflow of the suspect screening analysis, steps followed, parameters for feature prioritisation, confidence levels, and number of compounds detected in the samples

Figure 4 summarises the workflow used for compound identification with the following steps. (1) In the feature generation and prioritisation, the *m/z* traces determined by the *Deconvolution Plugin* yielded approximately 2500 compounds as potential candidates matched against the NIST library. To assure a high level of confidence in assignments, the prioritisation thresholds previously established (RSI > 750, RHRF > 90) were applied. Retention time alignment further refined this list to 1280 features with well-defined mass spectra and increased spectral-library match probability (confidence level 4). (2) In the tentative formula and structure assignment, the presence of the molecular ion or base peak ion within 5 ppm mass error was essential for tentative chemical formula assignment to the candidates (confidence level 3). In addition, the presence of other ions was manually evaluated in the mass spectra to confirm whether the observed fragmentation patterns were consistent with the proposed chemical structure (confidence level 2). The suspect screening methodology was also evaluated using a standard mixture containing all target compounds at a concentration of 40 ng mL^−1^, followed by the analysis of QCMs spiked at 40 ng g^−1^ dw for sediment and 50 ng g^−1^ dw for egg sample (confidence level 1). Table S4 shows the results for the *Deconvolution Plugin* automatic identification; among the 45 compounds studied, most of them were correctly identified. Nevertheless, the presence of some of them was not automatically confirmed probably due to the presence of matrix contents, which made more difficult the match with the spectral library. Among isomeric compounds (i.e. PCBs and PAHs), the given library names and their assigned chemical formulas were sometimes not correct, and thus, this was solved by manually assigning the correct name to these compounds according to the retention times and mass spectra from the tested standard solution. There is also evidence that the *Deconvolution Plugin* software reliably assigns a unique match from the NIST library when compound concentrations exceed 50 ng g^−1^ dw [31]. Below this threshold, manual identification and confirmation are strongly recommended. The performance of these approaches is inherently compound-dependent and is influenced by ionisation and ion fragmentation characteristics.

### Application of the suspect screening approach to the analysis of sediments and gull eggs from the Ebro Delta

The proposed suspect screening approach was then applied to characterise organic contaminants in environmental samples. From the five sediments, one of the most contaminated sediment samples (Flix 1) was selected for comprehensive data analysis, while from the four analysed eggs, one sample collected in 2019 was chosen. Among the compounds detected, PCB congeners were by far the dominant POPs detected in both matrices, and their full identification was a real challenge due to their multiple congener structures with common *m/z* values. Table 3 presents the compounds tentatively identified at confidence level 2, reporting retention time, number of chlorine atoms for PCBs, the most intense ions with their respective mass error values (in ppm), the NIST library hit compound name, and the RSI score (the RFRH score was set to > 90 for the displayed chemicals). In the Flix sediment, 88 compounds were identified, including 73 PCBs, and in the *Larus audouinii* egg, 43 compounds were detected, from which 33 were PCBs. The confirmation of PCBs relied on verifying that each NIST library match corresponded to the correct chlorination degree according to the chlorination level. This proposed suspect screening approach confirmed the correct assignment of all the PCBs in both matrices, in terms of chlorination level. Profiles differed greatly in the two samples; low-chlorinated PCBs were abundant in the sediment, but the eggs exhibited an increase from penta- to octa-chlorinated congeners. Moreover, the widespread occurrence of these PCBs is related to their high bioaccumulation potential, and this study has generated new data regarding the diversity of chlorinated chemicals present in the Flix hotspot pollution site.

**Table 3:**
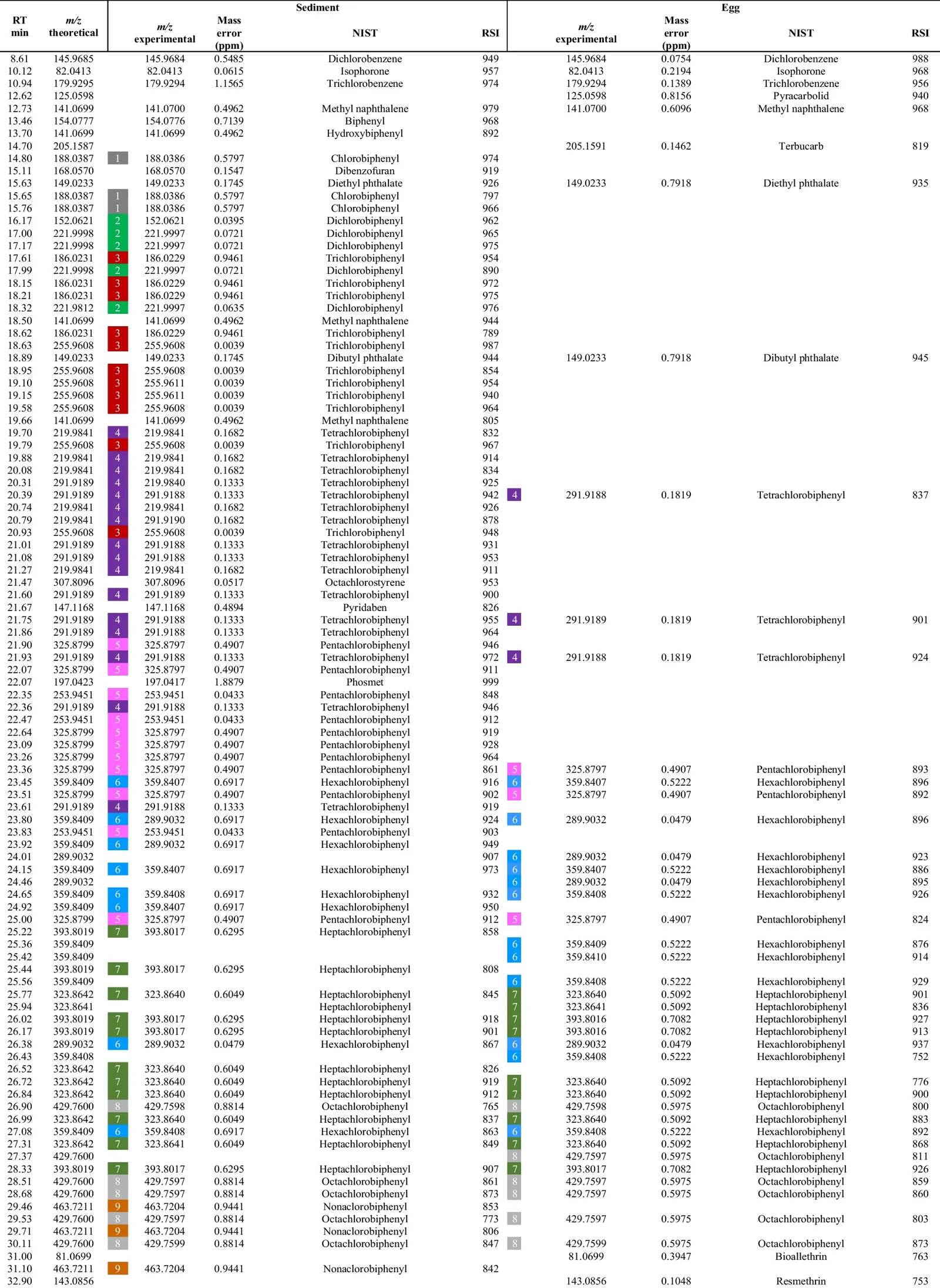
Identified compounds with the suspect approach. Rows refer to unique compounds and their retention time (min) and theoretical *m/z* values. For the sediment and for the gull egg, the information provided includes the number of chlorine atoms for PCBs detected, experimental *m/z* base peak, and mass error (in ppm), followed by its specific name given by the *Deconvolution Plugin* or, only for PCBs, the congener chlorination level generic name, and the Reversed Search Index (RSI) score given by the software. Empty columns are for non-detected compounds

Table S5 shows 15 additional compounds detected at confidence level 2 (confirmed by library spectrum match). Five compounds, such as biphenyl and dibenzofuran (industrial chemicals), octachlorostyrene, an unintended by-product of some electrolytic processes, and phosmet and pyridaben, two agrochemicals, were characterised only in the sediment. Pesticide bioallethrin, pyracarbolid, terbucarb, and resmethrin were identified only in the egg, and these chemicals are widely used in agriculture; the last one is employed against mosquitoes, and its presence matches their potential use in the Ebro Delta, a floodable area affected by these insects. Finally, six compounds were found in both matrices, indicating a widespread exposure. These compounds were hydroxybiphenyl, trichlorobenzene, dichlorobenzene, dibutyl phthalate and diethyl phthalate, and methyl naphthalene, related either to industrial uses as heat-transfer mediums or solvents, used as plastic additives, or employed for agrochemical applications.

Overall, the proposed suspect screening methodology enabled the detection of a broad range of organic contaminants, including legacy pollutants such as PCBs, agrochemicals, and plasticizers, and allowed the identification of industrial chemicals and by-products. These findings highlight the value of integrated targeted and suspect screening approaches to expand chemical coverage and improve confidence in compound identification to understand transport and accumulation of contaminants in the sediment-biota system. Nevertheless, further research is needed to enhance the characterisation and detection of non-routinely monitored organic pollutants, reinforcing the relevance of suspect screening analysis and non-target analysis for a comprehensive assessment of chemical burdens in bird populations.

## Conclusions

An analytical methodology based on GC–EI–Orbitrap–HRMS was developed (i) to screen and quantify 45 POPs and PAHs and (ii) to investigate the presence of additional organic contaminants in highly polluted sediments from Flix and in *Larus audouinii* eggs from the Ebro Delta Natural Park. Quality parameters of the developed analytical methodology demonstrated its sensitivity and selectivity, and the uncertainty was determined to prove its overall robustness. Target analysis revealed contrasting contamination profiles between sediments and the eggs. 4,4′-DDT was detected exclusively in sediments, whereas its metabolite 4,4′-DDE was mainly found only in eggs, indicating metabolization of 4,4′-DDT and biomagnification in biota. High-chlorinated PCBs predominated in eggs rather than sediments, with slightly higher concentrations in egg samples collected in 2012 compared with 2019. The suspect screening approach, conducted through *Deconvolution Plugin* by filtering the spectra searched against the NIST exact mass spectral library (RSI > 750, and RHRF > 90), substantially reduced the number of putative candidates. From an initial set of 1,280 features, a total of 88 compounds in the sediments and 43 compounds in *Larus audouinii* eggs were identified and characterised. PCBs were the most ubiquitous contaminants in both matrices and were associated to the historical industrial pollution from the Flix chlor-alkali plant, demonstrating their transport downstream to the Ebro Delta, their bioaccumulation in gulls, and their broader impact on other species sharing habitat. It was also observed that sediment removal activities were associated with a decrease in concentrations in gulls. Additional contaminants identified in the analysed samples included agrochemicals, associated with rice cultivation and industrial chemicals, and plasticizers linked to regional industrial activities. The integration of targeted analysis with suspect screening markedly expanded the range of detectable organic contaminants and improved confidence in compound identification. The capacity to enlarge the number of organic compounds will provide more data on the impact of pollution hotspots in natural areas of ecological interest. Overall, this study adds knowledge on highly bioaccumulative and persistent compounds and strengthens the basis for evaluating their toxicological relevance in wildlife.

## Supplementary Information

Below is the link to the electronic supplementary material.

Supplementary file1 (DOCX 890 KB)

## Data Availability

Data will be made available on request.
