# Supplementary material for: Comprehensive characterisation of organic pollutants in high-polluted sediments and gull eggs using a broad-scope gas chromatography–Orbitrap methodology
Source: Anal Bioanal Chem. 2026 May 9;418(18):6059–73. doi: 10.1007/s00216-026-06532-z (PMC13541997; doi:10.1007/s00216-026-06532-z)
Supplement: Supplementary file 1 [file 216_2026_6532_MOESM1_ESM.docx]

**Supplementary Material**

Comprehensive characterization of organic pollutants in high-polluted sediments and gull eggs using a broad-scope gas chromatography–Orbitrap methodology

Bernat Oró-Nolla ^1,2,^ *, Francisco Javier Santos ^3^, Albert Bertolero ^4^, Sílvia Lacorte ^1,^ *

^1^ Department of Environmental Chemistry, IDAEA-CSIC, Jordi Girona 18-26, 08034 Barcelona, Catalonia, Spain

^2^ Department of Geosciences, University of Tübingen, Schnarrenbergstraße 94-96, 72076, Tübingen, Germany

^3^ Department of Chemical Engineering and Analytical Chemistry, University of Barcelona, Martí i Franquès 1, 08028 Barcelona, Catalonia, Spain

^4^ Associació Ornitològica Picampall de les Terres de l’Ebre, Trinquet 8, 43580 Deltebre, Spain

* Corresponding authors: bernat.nolla@uni-tuebingen.de and slbqam@cid.csic.es

**Table of contents**

**Text S1.** Chemical and reagents………………………………………………………………………...	2

**Table S1.** Target pollutants and their associated internal standards……………………………………	3

**Table S2.** Target halogenated persistent organic pollutants and PAHs ordered by retention time, indicating quantifier ion (Q1, with 100% if not indicated) and qualifier ions (q2 and q3) for confirmation with their mass accuracy errors by GC–EI–Orbitrap–HRMS analysis…………………………………	4

**Table S3.** Uncertainty components and combined uncertainty for sediments and gull eggs…………...	6

**Table S4.** Target compounds identified by the Deconvolution Plugin for a 40 ng mL^-1^ standard solution and the sediment and egg QCMs, using the proposed suspect screening approach………………………8

**Table S5.** Name and uses (from PubChem and Hazardous Substances Data Bank (HSDB)) of the organic pollutants identified in sediments and eggs through the suspect methodology…………………………10

**Fig S1.** Gas chromatography – high-resolution mass spectrometry (GC-HRMS) chromatogram of the total ion current (TIC) of a standard solution at 500 ng mL^-1^ for all the target analytes ………………12

**Text S1**. Chemicals and reagents

Standard Pesticide Mix1, containing aldrin, α-, β-, γ- and δ-hexachlorocyclohexane (HCH), α- and β-chlordane, 2,4′-DDD, 2,4′-DDE, 2,4′-DDT, 4,4′-DDD, 4,4′-DDE, 4,4′-DDT, dieldrin, heptachlor, heptachlor exo- and endo- epoxides, hexachlorobenzene (HCB), isodrin, methoxychlor, mirex, and oxychlordane at a concentration of 10 µg mL^-1^ and the PCB congeners: 28, 52, 101, 138, 153 and 180, at 1 µg mL^-1^ in toluene were acquired from AccuStandard (New Haven, CT, USA). Standard PCB Mix3, containing PCB congeners: 28, 52, 101, 118, 138, 153 and 180, at 10 µg mL^-1^ in isooctane were obtained from Dr. Ehrenstorfer, Augsburg, Germany. Individual standard solution of hexachlorobutadiene (HCBu) at 5000 µg mL^-1^ and pentachlorobenzene (PeCB) (solid, 98% purity) were acquired from Sigma-Aldrich (Darmstadt, Germany and St. Louis, MO, USA). A solution of polycyclic aromatic hydrocarbons (PAHs) mix at 200 µg mL^-1^ in toluene containing naphthalene, acenaphthylene, acenaphthene, fluorene, phenanthrene, anthracene, fluoranthene, pyrene, 1,2-benzanthracene, chrysene, benzo[b]fluoranthene, benzo[k]fluoranthene, benzo[a]pyrene, indeno[1,2,3-cd]pyrene, dibenz[a,h]anthracene, benzo[g,h,i]perylene, were purchased from AccuStandard, (New Haven, CT, USA). Finally, a standard mixture of all the target compounds (OCPs, PCBs, PBDEs and PAHs) was prepared at 1 µg μL^-1^ in isooctane.

As internal standards, the following compounds were employed: ^13^C_6_–HCB at 100 µg mL^-1^ in acetone and ^13^C_12_–4,4’–DDE at 100 µg mL^-1^ in nonane were purchased from Cambridge Isotope Laboratories, Inc. (CIL, Andover, MA, USA). Standard solutions of PCB 65 at 35 µg mL^-1^ and PCB 209 at 10 µg mL^-1^ in hexane were supplied by Dr Ehrenstofer, Augsburg, Germany. Isotope labelled PAHs solution mix at 4000 µg mL^-1^ containing ^2^H_10_-acenaphthene, ^2^H_10_-phenanthrene, ^2^H_12_-chrysene, and ^2^H_12_-perylene, were obtained from Sigma-Aldrich (Darmstadt, Germany and St. Louis, MO, USA). A standard mixture containing all internal standards was prepared at 1 µg mL^-1^ in isooctane.

**Table S1**. Target pollutants and their associated internal standards.

| Compound | Molecular Formula | CAS Num. | Internal standards |
| --- | --- | --- | --- |
| Hexachlorobutadiene (HCBu) | C_4_Cl_6_ | 87-68-3 | ^13^C_6_-Hexachlorobenzene |
| Acenaphthylene | C_12_H_8_ | 208-96-8 | ^2^H_10_-Acenaphtene |
| Acenaphthene | C_12_H_10_ | 83-32-9 | ^2^H_10_-Acenaphtene |
| Pentachlorobenzene (PeCB) | C_6_HCl_5_ | 608-93-5 | ^13^C_6_-Hexachlorobenzene |
| Fluorene | C_13_H_10_ | 86-73-7 | ^2^H_10_-Acenaphtene |
| α-HCH | C_6_H_6_Cl_6_ | 319-84-6 | ^13^C_6_-Hexachlorobenzene |
| Hexachlorobenzene (HCB) | C_6_Cl_6_ | 118-74-1 | ^13^C_6_-Hexachlorobenzene |
| β-HCH | C_6_H_6_Cl_6_ | 319-85-7 | ^13^C_6_-Hexachlorobenzene |
| 𝛾-HCH | C_6_H_6_Cl_6_ | 58-89-9 | ^13^C_6_-Hexachlorobenzene |
| Phenanthrene | C_14_H_10_ | 85-01-8 | ^2^H_10_-Phenanthrene |
| Anthracene | C_14_H_10_ | 120-12-7 | ^2^H_10_-Phenanthrene |
| δ-HCH | C_6_H_6_Cl_6_ | 319-86-8 | ^13^C_6_-Hexachlorobenzene |
| PCB28 | C_12_H_7_Cl_3_ | 7012-37-5 | PCB 65 |
| Heptachlor | C_10_H_5_Cl_7_ | 76-44-8 | ^13^C_12_-4,4'-DDE |
| PCB52 | C_12_H_6_Cl_4_ | 68194-04-7 | PCB 65 |
| Aldrin | C_12_H_8_Cl_6_ | 309-00-2 | ^13^C_12_-4,4'-DDE |
| Isodrin | C_12_H_8_Cl_6_ | 465-73-6 | ^13^C_12_-4,4'-DDE |
| Heptachlorepoxide | C_10_H_5_Cl_7_O | 1024-57-3 | ^13^C_12_-4,4'-DDE |
| Oxychlordane | C_5_Cl_6_ | 77-47-4 | ^13^C_12_-4,4'-DDE |
| Fluoranthene | C_16_H_10_ | 206-44-0 | ^2^H_10_-Phenanthrene |
| 2,4′-DDE | C_14_H_8_Cl_4_ | 3424-82-6 | ^13^C_12_-4,4'-DDE |
| α-Chlordane | C_10_H_6_Cl_8_ | 5103-71-9 | ^13^C_12_-4,4'-DDE |
| PCB101 | C_12_H_5_Cl_5_ | 38380-01-7 | ^13^C_12_-4,4'-DDE |
| β-Chlordane | C_10_H_6_Cl_8_ | 5103-74-2 | ^13^C_12_-4,4'-DDE |
| Pyrene | C_16_H_10_ | 129-00-0 | ^2^H_10_-Phenanthrene |
| 4,4′-DDE | C_14_H_8_Cl_4_ | 72-55-9 | ^13^C_12_-4,4'-DDE |
| 2,4′-DDD | C_14_H_10_Cl_4_ | 53-19-0 | ^13^C_12_-4,4'-DDE |
| Dieldrin | C_12_H_8_Cl_6_O | 60-57-1 | ^13^C_12_-4,4'-DDE |
| PCB118 | C_12_H_5_Cl_5_ | 57465-28-8 | PCB 65 |
| 4,4′-DDD | C_14_H_10_Cl_4_ | 72-54-8 | ^13^C_12_-4,4'-DDE |
| 2,4′-DDT | C_14_H_9_Cl_5_ | 789-02-6 | ^13^C_12_-4,4'-DDE |
| PCB153 | C_12_H_4_Cl_6_ | 35065-28-2 | PCB 209 |
| 4,4′-DDT | C_14_H_9_Cl_5_ | 50-29-3 | ^13^C_12_-4,4'-DDE |
| PCB138 | C_12_H_4_Cl_6_ | 35065-28-2 | PCB 209 |
| Methoxychlor | C_16_H_15_Cl_3_O_2_ | 72-43-5 | ^13^C_12_-4,4'-DDE |
| 1,2-Benzanthracene | C_18_H_12_ | 56-55-3 | ^2^H_12_-Chrysene |
| Chrysene | C_18_H_12_ | 218-01-9 | ^2^H_12_-Chrysene |
| PCB180 | C_12_H_3_Cl_7_ | 52663-68-0 | PCB 209 |
| Mirex | C_10_Cl_12_ | 2385-85-5 | ^13^C_12_-4,4'-DDE |
| Benzo[b]fluoranthene | C_20_H_12_ | 205-99-2 | ^2^H_12_-Perylene |
| Benzo[k]fluoranthene | C_20_H_12_ | 207-08-9 | ^2^H_12_-Perylene |
| Benzo[a]pyrene | C_20_H_12_ | 50-32-8 | ^2^H_12_-Perylene |
| Indeno[1,2,3-cd]pyrene | C_22_H_12_ | 193-39-5 | ^2^H_12_-Perylene |
| Dibenz[a,h]anthracene | C_22_H_12_ | 53-70-3 | ^2^H_12_-Perylene |
| Benzo[g,h,i]perylene | C_22_H_12_ | 191-24-2 | ^2^H_12_-Perylene |

**Table S2**. Target halogenated persistent organic pollutants and PAHs ordered by retention time, indicating quantifier ion (Q1, with 100% if not indicated) and qualifier ions (q2 and q3) for confirmation with their mass accuracy errors by GC–EI–Orbitrap–HRMS analysis.

| Compound | Retention time  (min) | Quantifier ion Q1 (*m/z*) | Assigned ion Q1  (rel. abundance %) | Mass accuracy (ppm) | Qualifier ion q2 (*m/z*) | Assigned ion q2  (rel. abundance %) | Mass accuracy (ppm) | Qualifier ion q3 (*m/z*) | Assigned ion q3  (rel. abundance %) | Mass accuracy (ppm) |
| --- | --- | --- | --- | --- | --- | --- | --- | --- | --- | --- |
| HCBu | 11.34 | 224.8407 | C_4_Cl_4_^37^Cl | -1.07 | 226.8378 | C_4_Cl_3_^37^Cl_2_ (65) | -0.71 | 222.8437 | C_4_Cl_5_ (60) | -0.63 |
| Acenaphthylene | 14.35 | 152.0620 | C_12_H_8_ | -1.18 | 150.0465 | C_12_H_6_ (20) | -1.47 | 151.0546 | C_12_H_7_ (15) | 0.46 |
| Acenaphthene (^2^H_10_) | 14.65 | 162.1263 | C_12_D_9_ | -0.25 | 164.1404 | C_12_D_10_ (74) | -0.24 | 160.1122 | C_12_D_8_ (47) | -0.75 |
| Acenaphthene | 14.72 | 153.0698 | C_12_H_9_ | -0.85 | 154.0776 | C_12_H_10_ (60) | 0.45 | 152.0621 | C_12_H_8_ (53) | -0.59 |
| PeCB | 15.00 | 249.8486 | C_6_HCl_4_^37^Cl | -0.48 | 251.8456 | C_6_HCl_3_^37^Cl_2_ (64) | -0.40 | 247.8516 | C_6_HCl_5_ (62) | -0.48 |
| Fluorene | 15.89 | 165.0698 | C_13_H_9_ | 0.30 | 166.0775 | C_13_H_10_ (64) | 0.18 | 163.0543 | C_13_H_7_ (17) | 0.31 |
| α-HCH | 17.13 | 180.9373 | C_6_H_4_Cl_3_ | -0.50 | 182.9344 | C_6_H_4_Cl_2_^37^Cl (96) | -0.16 | 218.9111 | C_6_H_5_Cl_3_^37^Cl (36) | -1.19 |
| Hexachlorobenzene (^13^C_6_) | 17.24 | 289.8297 | ^13^C_6_Cl_5_^37^Cl | -0.14 | 291.8268 | ^13^C_6_Cl_4_^37^Cl_2_ (79) | -0.17 | 287.8327 | ^13^C_6_Cl_6_ (53) | -0.15 |
| HCB | 17.25 | 283.8097 | C_6_Cl_5_^37^Cl | -0.67 | 285.8067 | C_6_Cl_4_^37^Cl_2_ (81) | -0.56 | 281.8127 | C_6_Cl_6_ (55) | -0.78 |
| β-HCH | 17.66 | 180.9373 | C_6_H_4_Cl_3_ | -0.44 | 182.9344 | C_6_H_4_Cl_2_^37^Cl (96) | -0.05 | 218.9111 | C_6_H_5_Cl_3_^37^Cl (55) | -1.23 |
| 𝛾-HCH | 17.93 | 180.9373 | C_6_H_4_Cl_3_ | -0.44 | 182.9344 | C_6_H_4_Cl_2_^37^Cl (96) | -0.05 | 218.9111 | C_6_H_5_Cl_3_^37^Cl (55) | -1.19 |
| Phenanthrene (^2^H_10_) | 18.30 | 188.1403 | C_14_D_10_ | -0.96 | 184.1123 | C_14_D_8_ (18) | 0.11 | 186.1265 | C_14_H_6_ (15) | 0.48 |
| Phenanthrene | 18.37 | 178.0776 | C_14_H_10_ | -0.67 | 176.0621 | C_14_H_8_ (25) | -0.97 | 174.0465 | C_14_H_6_ (15) | 1.67 |
| Anthracene | 18.52 | 178.0777 | C_14_H_10_ | -0.51 | 176.0621 | C_14_H_8_ (24) | -0.91 | 174.0465 | C_14_H_6_ (15) | 1.84 |
| δ-HCH | 18.55 | 180.9374 | C_6_H_4_Cl_3_ | -0.33 | 182.9344 | C_6_H_4_Cl_2_^37^Cl (96) | 0.11 | 218.9111 | C_6_H_5_Cl_3_^37^Cl (36) | -1.05 |
| PCB 28 | 19.35 | 255.9608 | C_12_H_7_Cl_3_ | 1.02 | 257.9579 | C_12_H_7_Cl_2_^37^Cl (97) | 1.09 | 186.0231 | C_12_H_7_Cl (65) | -0.91 |
| Heptachlor | 19.78 | 271.8098 | C_5_Cl_5_^37^Cl | 0.01 | 273.8069 | C_5_Cl_4_^37^Cl_2_ (85) | 0.11 | 269.8128 | C_5_Cl_6_ (53) | -0.04 |
| PCB 52 | 20.19 | 291.9190 | C_12_H_6_Cl_3_^37^Cl | -0.31 | 219.9842 | C_12_H_6_Cl_2_ (95) | 0.23 | 289.9219 | C_12_H_6_Cl_4_ (69) | 0.45 |
| PCB 65 | 20.44 | 291.9189 | C_12_H_6_Cl_3_^37^Cl | -0.65 | 289.9219 | C_12_H_6_Cl_4_ (80) | 0.24 | 219.9841 | C_12_H_6_Cl_2_ (54) | 0.05 |
| Aldrin | 20.75 | 262.8566 | C_7_H_2_Cl_4_^37^Cl | 0.11 | 264.8537 | C_7_H_2_Cl_3_^37^Cl_2_ (65) | 0.34 | 260.8595 | C_7_H_2_Cl_5_ (62) | 0.35 |
| Isodrin | 21.57 | 192.9374 | C_7_H_4_Cl_3_ | 0.88 | 194.9344 | C_7_H_4_Cl_2_^37^Cl (98) | 0.92 | 262.8566 | C_7_H_2_Cl_4_^37^Cl (68) | 0.68 |
| Heptachlor epoxide | 21.77 | 352.8437 | C_10_H_5_OCl_5_^37^Cl | -1.30 | 354.8408 | C_10_H_5_OCl_4_^37^Cl_2_ (90) | -1.13 | 81.0336 | C_5_H_5_O (53) | -1.36 |
| Oxychlordane | 21.88 | 252.8956 | C_9_H_3_Cl_3_^37^Cl | -1.42 | 236.8409 | C_5_Cl_4_^37^Cl (94) | 0.63 | 250.8985 | C_9_H_3_Cl_4_ (74) | 0.68 |
| Fluoranthene | 22.06 | 202.0776 | C_16_H_10_ | 1.68 | 200.0620 | C_16_H_8_ (26) | 0.55 | 201.0700 | C_16_H_9_ (20) | 1.34 |
| 2,4'-DDE | 22.38 | 245.9999 | C_14_H_8_Cl_2_ | 0.49 | 247.9969 | C_14_H_8_Cl^37^Cl (64) | 0.52 | 317.9345 | C_14_H_8_Cl_3_^37^Cl (32) | 0.38 |
| α-Chlordane | 22.41 | 372.8254 | C_10_H_6_Cl_6_^37^Cl | 0.08 | 374.8225 | C_10_H_6_Cl_5_^37^Cl_2_ (95) | 0.24 | 236.8409 | C_5_Cl_4_^37^Cl (55) | 0.30 |
| PCB 101 | 22.50 | 325.8798 | C_12_H_5_Cl_4_^37^Cl | -0.77 | 253.9452 | C_12_H_5_Cl_3_ (65) | 0.32 | 327.8769 | C_12_H_5_Cl_3_^37^Cl_2_ (65) | -0.49 |
| β-Chlordane | 22.77 | 372.8254 | C_10_H_6_Cl_6_^37^Cl | 0.08 | 374.8225 | C_10_H_6_Cl_5_^37^Cl_2_ (97) | 0.24 | 236.8409 | C_5_Cl_4_^37^Cl (85) | 0.13 |
| Pyrene | 22.82 | 202.0776 | C_1_6H_10_ | 1.73 | 200.0621 | C_16_H_8_ (26) | 1.00 | 201.0700 | C_16_H_9_ (20) | 1.34 |
| 4,4'-DDE (^13^C_12_) | 23.26 | 258.0401 | C_2_^13^C_12_H_8_Cl_2_ | 0.35 | 260.0372 | C_2_^13^C_12_H_8_Cl^37^Cl (65) | 0.42 | 329.9747 | C_2_^13^C_12_H_8_Cl_3_^37^Cl (51) | -0.21 |
| 4,4'-DDE | 23.27 | 245.9999 | C_14_H_8_Cl_2_ | 0.53 | 247.9969 | C_14_H_8_Cl^37^Cl (64) | 0.52 | 317.9345 | C_14_H_8_Cl_3_^37^Cl (30) | 0.38 |
| 2,4'-DDD | 23.48 | 235.0077 | C_13_H_9_Cl_2_ | 0.21 | 237.0047 | C_13_H_9_Cl^37^Cl (65) | 0.25 | 165.0700 | C_13_H_9_ (42) | 0.55 |
| Dieldrin | 23.57 | 262.8563 | C_7_H_2_Cl_4_^37^Cl (90) | -0.30 | 79.0543 | C_6_H_7_ (100) | 1.39 | 236.8409 | C_5_Cl_4_^37^Cl (45) | 0.68 |
| PCB 118 | 24.25 | 325.8799 | C_12_H_5_Cl_4_^37^Cl | -0.58 | 327.8769 | C_12_H_5_Cl_3_^37^Cl_2_ (75) | -0.58 | 253.9453 | C_12_H_5_Cl_3_ (65) | -0.35 |
| 4,4'-DDD | 24.47 | 235.0077 | C_13_H_9_Cl_2_ | 0.34 | 237.0048 | C_13_H_9_Cl^37^Cl (65) | 0.46 | 165.0700 | C_13_H_9_ (42) | 0.67 |
| 2,4'-DDT | 24.57 | 235.0077 | C_13_H_9_Cl_2_ | 0.26 | 237.0048 | C_13_H_9_Cl^37^Cl (65) | 0.46 | 165.0700 | C_13_H_9_ (35) | 0.55 |
| PCB 153 | 24.83 | 359.8410 | C_12_H_4_Cl_5_^37^Cl | 0.58 | 361.8378 | C_12_H_4_Cl_4_^37^Cl_2_ (86) | 0.01 | 289.9034 | C_12_H_4_Cl_3_^37^Cl (55) | -0.21 |
| 4,4'-DDT | 25.56 | 235.0078 | C_13_H_9_Cl_2_ | 0.60 | 237.0048 | C_13_H_9_Cl^37^Cl (65) | 0.46 | 165.0700 | C_13_H_9_ (42) | 0.73 |
| PCB 138 | 25.61 | 359.8410 | C_12_H_4_Cl_5_^37^Cl | 0.67 | 361.8381 | C_12_H_4_Cl_4_^37^Cl_2_ (81) | 0.75 | 289.9034 | C_12_H_4_Cl_3_^37^Cl (55) | 0.10 |
| Methoxychlor | 27.11 | 227.1068 | C_15_H_15_O_2_ | 0.92 | 228.1101 | C_13_H_19_O^37^Cl (16) | 0.48 | 274.0758 | C_16_H_15_O_2_Cl (10) | -0.33 |
| 1,2-Benzanthracene | 27.17 | 228.0934 | C_18_H_12_ | 0.44 | 226.0779 | C_18_H_10_ (35) | -0.40 | 224.0622 | C_18_H_8_ (25) | -0.49 |
| Chrysene (^2^H_12_) | 27.20 | 240.1687 | C_18_D_12_ | -0.04 | 236.1406 | C_18_D_10_ (30) | 0.38 | 238.1547 | C_18_D_11_ (20) | 0.67 |
| Chrysene | 27.30 | 228.0935 | C_18_H_12_ | 0.53 | 226.0779 | C_18_H_10_ (35) | -0.49 | 224.0622 | C_18_H_8_ (31) | -0.67 |
| PCB 180 | 27.50 | 393.8020 | C_12_H_3_Cl_6_^37^Cl | 0.46 | 395.7991 | C_12_H_3_Cl_5_^37^Cl_2_ (95) | 0.45 | 323.8643 | C_12_H_3_Cl_4_^37^Cl (50) | 0.37 |
| Mirex | 28.87 | 271.8099 | C_5_Cl_5_^37^Cl | 0.59 | 273.8070 | C_5_Cl_4_^37^Cl_2_ (80) | 0.69 | 236.8409 | C_5_Cl_4_^37^Cl (60) | 0.55 |
| Benzo[b]fluoranthene | 31.01 | 252.0936 | C_20_H_12_ | 0.63 | 250.0780 | C_20_H_10_ (27) | -0.24 | 248.0624 | C_20_H_8_ (20) | -0.36 |
| Benzo[k]fluoranthene | 31.11 | 252.0936 | C_20_H_12_ | 0.63 | 250.0780 | C_20_H_10_ (27) | -0.24 | 248.0623 | C_20_H_8_ (20) | -0.44 |
| PCB 209 | 31.92 | 497.6822 | C_12_Cl_8_^37^Cl_2_ | 0.22 | 499.6792 | C_12_Cl_7_^37^Cl_3_ (85) | 0.20 | 495.6854 | C_12_Cl_9_^37^Cl (73) | 0.67 |
| Benz[a]pyrene | 32.09 | 252.0936 | C_20_H_12_ | 0.56 | 250.0779 | C_20_H_10_ (27) | -0.48 | 248.0620 | C_20_H_8_ (20) | -1.65 |
| Perylene (^2^H_12_) | 32.28 | 264.1689 | C_20_D_12_ | 0.91 | 260.1404 | C_20_D_10_ (90) | -0.46 | 262.1545 | C_20_D_11_ (55) | -0.42 |
| Indeno[1,2,3-cd]pyrene | 35.82 | 276.0936 | C_22_H_12_ | 1.34 | 274.0779 | C_22_H_10_ (30) | -0.22 | 275.0854 | C_22_H_11_ (20) | 0.51 |
| Dibenz[a,h]anthracene | 35.93 | 278.1093 | C_22_H_14_ | 1.51 | 276.0934 | C_22_H_12_ (5) | 0.47 | 274.0782 | C_22_H_10_ (20) | 0.91 |
| Benzo[ghi]perylene | 36.78 | 276.0936 | C_22_H_12_ | 1.45 | 274.0778 | C_22_H_10_ (30) | -0.33 | 275.0856 | C_22_H_11_ (20) | 1.26 |

**Table S3**. Uncertainty components for bias and precision and combined uncertainty for sediments and eggs, respectively.

| Compounds | Sediments | | | | | Eggs | | | | |
| --- | --- | --- | --- | --- | --- | --- | --- | --- | --- | --- |
|  | Repeatability  (n=5, RSD%) | Reproducibility (n=5, RSD%) | u' % (bias) | u' % (precision) | u' % | Repeatability  (n=5, RSD%) | Reproducibility  (n=5, RSD%) | u' % (bias) | u' % (precision) | u' % |
| HCBu | 1.0 | 21 | 21 | 22 | 30 | 12 | 14 | 18 | 11 | 21 |
| Acenaphthylene | 7.3 | 0.4 | 6.9 | 7.6 | 10 | 4.0 | 3.1 | 6.0 | 4.4 | 7.4 |
| Acenaphthene | 3.2 | 0.2 | 3.4 | 1.3 | 3.6 | 4.2 | 0.4 | 4.1 | 3.8 | 5.6 |
| PeCB | 10 | 3.3 | 14 | 10 | 17 | 2.4 | 0.9 | 17 | 2.5 | 17 |
| Fluorene | 6.5 | 3.3 | 12 | 5.8 | 13 | 8.1 | 12 | 18 | 2.6 | 18 |
| α-HCH | 6.6 | 6.1 | 5.0 | 5.5 | 7.4 | 3.9 | 4.6 | 6.5 | 5.1 | 8.3 |
| HCB | 7.7 | 0.9 | 16 | 5.9 | 17 | 10 | 12 | 19 | 11 | 22 |
| β-HCH | 8.7 | 8.2 | 7.6 | 6.9 | 10 | 5.2 | 5.1 | 6.5 | 7.1 | 9.6 |
| 𝛾-HCH | 6.2 | 6.9 | 4.6 | 5.1 | 6.9 | 3.2 | 3.7 | 4.3 | 4.2 | 6.0 |
| Phenanthrene | 2.3 | 2.1 | 4.5 | 4.8 | 6.5 | 9.4 | 4.4 | 18 | 7.9 | 19 |
| Anthracene | 4.1 | 0.9 | 0.8 | 0.7 | 1.1 | 3.3 | 4.5 | 15 | 1.7 | 15 |
| δ-HCH | 6.9 | 8.3 | 5.7 | 6.3 | 8.5 | 5.1 | 4.5 | 6.8 | 5.2 | 8.5 |
| PCB28 | 3.7 | 0.4 | 15 | 1.8 | 15 | 2.5 | 1.2 | 15 | 1.2 | 15 |
| Heptachlor | 4.4 | 0.1 | 20 | 1.6 | 20 | 2.2 | 0.8 | 11 | 1.8 | 11 |
| PCB52 | 4.0 | 0.1 | 7.9 | 1.6 | 8.1 | 2.0 | 0.9 | 6.9 | 1.4 | 7.1 |
| Aldrin | 7.4 | 7.6 | 31 | 6.6 | 32 | 2.8 | 2.6 | 12 | 0.2 | 12 |
| Isodrin | 15 | 18 | 58 | 13 | 59 | 2.0 | 0.4 | 3.9 | 4.3 | 5.8 |
| Heptachlorepoxide | 7.4 | 6.0 | 51 | 6.4 | 52 | 7.6 | 12 | 38 | 13 | 40 |
| Oxychlordane | 4.9 | 2.8 | 1.9 | 1.9 | 2.7 | 3.9 | 1.4 | 13 | 3.3 | 13 |
| Fluoranthene | 4.3 | 1.3 | 8.2 | 4.2 | 9.2 | 4.4 | 4.8 | 14 | 2.2 | 14 |
| 2,4′-DDE | 4.0 | 0.2 | 11 | 1.1 | 11 | 3.1 | 2.8 | 12 | 0.6 | 12 |
| α-Chlordane | 4.4 | 0.3 | 3.5 | 1.0 | 3.6 | 1.9 | 1.1 | 8.1 | 1.3 | 8.2 |
| PCB101 | 4.8 | 1.4 | 4.2 | 2.6 | 4.9 | 3.8 | 5.0 | 1.9 | 1.9 | 2.7 |
| β-Chlordane | 4.4 | 1.6 | 6.2 | 0.9 | 6.3 | 3.6 | 2.8 | 11 | 1.0 | 11 |
| Pyrene | 4.7 | 4.6 | 14 | 4.0 | 15 | 5.9 | 5.0 | 14 | 4.9 | 14 |
| 4,4′-DDE | 4.1 | 0.1 | 10 | 1.4 | 10 | 4.4 | 3.6 | 12 | 3.2 | 12 |
| 2,4′-DDD | 3.5 | 0.2 | 22 | 1.8 | 22 | 1.9 | 1.3 | 27 | 2.5 | 27 |
| Dieldrin | 4.1 | 1.0 | 87 | 2.3 | 87 | 8.0 | 10 | 67 | 20 | 70 |
| PCB118 | 4.4 | 0.4 | 8.4 | 1.8 | 8.5 | 3.0 | 3.2 | 5.8 | 0.9 | 5.8 |
| 4,4′-DDD | 3.7 | 0.2 | 26 | 1.1 | 26 | 3.8 | 2.8 | 27 | 3.0 | 27 |
| 2,4′-DDT | 5.4 | 1.0 | 16 | 4.0 | 16 | 2.4 | 0.1 | 12 | 2.9 | 12 |
| PCB153 | 4.1 | 0.8 | 3.2 | 2.5 | 4.1 | 3.9 | 4.0 | 2.5 | 2.1 | 3.3 |
| 4,4′-DDT | 9.5 | 4.2 | 23 | 17 | 28 | 4.3 | 1.5 | 15 | 16 | 21 |
| PCB138 | 4.2 | 0.2 | 1.9 | 1.0 | 2.2 | 3.9 | 3.3 | 2.6 | 1.9 | 3.2 |
| Methoxychlor | 6.6 | 4.4 | 72 | 10 | 73 | 14 | 19 | 62 | 19 | 65 |
| 1,2-Benzanthracene | 1.8 | 1.2 | 21 | 3.0 | 21 | 3.6 | 2.9 | 9.6 | 1.6 | 9.7 |
| Chrysene | 4.1 | 0.1 | 16 | 1.3 | 16 | 3.0 | 2.9 | 2.8 | 1.0 | 2.9 |
| PCB180 | 4.2 | 0.7 | 6.1 | 1.2 | 6.2 | 3.6 | 3.6 | 6.9 | 2.1 | 7.2 |
| Mirex | 5.2 | 0.2 | 3.3 | 3.5 | 4.9 | 3.8 | 0.7 | 3.5 | 3.8 | 5.2 |
| Benzo(b)fluoranthene | 3.6 | 3.9 | 10 | 3.9 | 11 | 3.2 | 0.2 | 3.2 | 2.5 | 4.0 |
| Benzo(k)fluoranthene | 3.3 | 3.3 | 20 | 3.1 | 20 | 3.1 | 0.1 | 10 | 2.2 | 10 |
| Benzo(a)pyrene | 4.7 | 3.2 | 67 | 8.3 | 68 | 21 | 25 | 35 | 28 | 45 |
| Indeno(1,2,3-cd)pyrene | 6.4 | 5.7 | 8.8 | 6.6 | 11 | 5.0 | 7.8 | 19 | 7.9 | 21 |
| Dibenz(a,h)anthracene | 6.8 | 7.6 | 18 | 16 | 24 | 4.8 | 5.1 | 13 | 14 | 19 |
| Benzo(g,h,i)perylene | 15 | 19 | 91 | 16 | 92 | 14 | 19 | 20 | 13 | 24 |

**Table S4**. Target compounds identified by the *Deconvolution Plugin* for a standard solution and the sediment and egg QCMs, using the proposed suspect screening approach.

|  |  | Standard solution ^(a)^ | | Sediment QCM ^(b)^ | | Egg QCM ^(c)^ | |
| --- | --- | --- | --- | --- | --- | --- | --- |
| Compound name | Qualifier ion (*m/z*) | RSI | RHRF  Score | RSI | RHRF  Score | RSI | RHRF  Score |
| HCBu | 224.8410 | 997 | 97.7 | 969 | 99.6 | 963 | 75.9 |
| Acenaphthylene | 152.0622 | 996 | 94.6 | 981 | 48.6 | n.c. | n.c. |
| Acenaphthene | 153.0699 | 996 | 95.4 | 984 | 81.1 | 963 | 79.9 |
| PeCB | 249.8487 | 993 | 91.8 | 962 | 88.4 | 935 | 81.0 |
| Fluorene | 165.0698 | 996 | 91.4 | 983 | 91.3 | 961 | 92.2 |
| α-HCH | 180.9372 | 982 | 94.4 | 925 | 94.5 | 867 | 95.0 |
| HCB | 283.8099 | 970 | 25.2 | 942 | 44.5 | n.c. | n.c. |
| β-HCH | 180.9372 | 982 | 96.0 | 913 | 90.0 | 856 | 86.3 |
| 𝛾-HCH | 180.9372 | 986 | 92.5 | 880 | 95.3 | 809 | 100 |
| Phenanthrene | 178.0777 | 994 | 97.3 | 985 | 98.2 | 972 | 99.4 |
| Anthracene | 178.0777 | 989 | 97.1 | 972 | 87.8 | 935 | 63.8 |
| δ-HCH | 180.9372 | 979 | 94.8 | 917 | 93.4 | 859 | 95.1 |
| PCB28 | 255.9608 | 992 | 92.4 | 956 | 96.7 | 923 | 96.9 |
| Heptachlor | 271.8098 | 948 | 98.0 | 826 | 99.0 | n.c. | n.c. |
| PCB52 | 219.9841 | 990 | 92.6 | 941 | 96.0 | 920 | 94.9 |
| Aldrin | 262.8565 | 947 | 98.9 | 773 | 95.6 | 704 | 90.9 |
| Isodrin | 192.9372 | 953 | 97.5 | 724 | 98.1 | 726 | 100 |
| Heptachlor epoxide | 352.8442 | 937 | 95.7 | 701 | 87.6 | n.c. | n.c. |
| Oxychlordane | 252.8959 | 939 | 58.4 | 687 | 95.5 | n.c. | n.c. |
| Fluoranthene | 202.0772 | 993 | 96.5 | 976 | 98.3 | 945 | 40.4 |
| 2,4′-DDE | 245.9997 | 976 | 92.6 | 916 | 96.1 | 859 | 97.1 |
| α-Chlordane | 372.8254 | 918 | 97.6 | 835 | 99.9 | 758 | 91.5 |
| PCB101 | 325.8798 | 979 | 90.0 | 953 | 91.3 | 935 | 93.4 |
| β-Chlordane | 372.8254 | 723 | 97.4 | 784 | 99.0 | 764 | 77.0 |
| Pyrene | 202.0772 | 991 | 94.1 | 973 | 98.1 | 947 | 100 |
| 4,4′-DDE | 245.9997 | 951 | 94.2 | 871 | 96.1 | 835 | 97.0 |
| 2,4′-DDD | 235.0076 | 973 | 97.3 | 948 | 98.7 | 917 | 99.3 |
| Dieldrin | 79.0542 | 867 | 97.0 | n.c. | n.c. | n.c. | n.c. |
| PCB118 | 325.8798 | 970 | 90.6 | 948 | 92.2 | 937 | 94.2 |
| 4,4′-DDD | 235.0076 | 909 | 98.4 | 890 | 98.4 | 873 | 100 |
| 2,4′-DDT | 235.0076 | 908 | 93.6 | 937 | 98.5 | 910 | 100 |
| PCB153 | 359.8410 | 965 | 92.7 | 908 | 94.3 | 862 | 96.2 |
| 4,4′-DDT | 235.0076 | 908 | 99.6 | 870 | 100 | 843 | 100 |
| PCB138 | 359.8410 | 961 | 93.1 | 901 | 94.4 | 845 | 88.9 |
| Methoxychlor | 227.1066 | 836 | 99.6 | n.c. | n.c. | n.c. | n.c. |
| 1,2-Benzanthracene | 228.0933 | 983 | 97.7 | 956 | 98.3 | 929 | 100 |
| Chrysene | 228.0933 | 982 | 97.4 | 951 | 97.9 | 935 | 100 |
| PCB180 | 393.8019 | 927 | 86.8 | 853 | 88.9 | 811 | 84.8 |
| Mirex | 271.8097 | 920 | 96.1 | 806 | 92.6 | 808 | 97.2 |
| Benzo[b]fluoranthene | 252.0934 | 956 | 99.3 | 958 | 98.1 | 847 | 100 |
| Benzo[k]fluoranthene | 252.0934 | 945 | 99.3 | 957 | 97.9 | 916 | 100 |
| Benzo[a]pyrene | 252.0934 | 924 | 100 | n.c. | n.c. | n.c. | n.c. |
| Indeno[1,2,3-cd]pyrene | 276.0934 | 807 | 100 | 919 | 100 | 920 | 89.2 |
| Dibenz[a,h]anthracene | 276.0934 | 814 | 98.9 | 864 | 100 | 838 | 56.4 |
| Benzo[ghi]perylene | 276.0934 | 856 | 100 | n.c. | n.c. | n.c. | n.c. |

(a) Concentration of all the compounds at 40 ng mL^-1^.
(b) Sediment QCM spiked at 40 ng g^-1^ dry weight.
(c) Egg QCM spiked at 50 ng g^-1^ dry weight.

n.c.: not confirmed

**Table S5**. Name and uses (from PubChem and Hazardous Substances Data Bank (HSDB)) of the organic pollutants identified in sediments and eggs through the suspect methodology.

| Name | Uses | Matrix |
| --- | --- | --- |
| Biphenyl (CAS 68409-73-4) | Industrial chemistry, organic syntheses, heat transfer fluids, dye carriers, food preservatives, as an intermediate for polychlorinated biphenyls, and as a fungistatic in the packaging of citrus fruits | Sediments |
| Dibenzofuran (CAS 132-64-9) | - Industrial chemistry, as a component of heat-transfer oils (with biphenyl); suitable as a carrier for dyeing and printing textiles (combined with methylnaphthalenes), derivatives serve as intermediates for naphthol AS and chromium complex dyes  - Agrochemistry, insecticide | Sediments |
| Octachlorostyrene (CAS 29082-74-4) | Industrial chemistry, unintended by-product of various industrial processes including electrolytic production of chlorine gas or magnesium, refining, and degassing in aluminium smelting, and chlorination and distillation to produce niobium and tantalum | Sediments |
| Phosmet (CAS 732-11-6) | Agrochemistry, non-systemic acaricide and insecticide for the control of lepidopterous larvae, aphids, suckers, fruit flies, and spider mites on pome fruit, stone fruit, citrus fruit, ornamentals, and vines; Colorado beetles on potatoes; boll weevils on cotton; olive moths and olive thrips on olives; blossom beetles on oilseed rape; leaf beetles and weevils on alfalfa; European corn borers on maize and sorghum | Sediments |
| Pyridaben (CAS 96489-71-3) | Agrochemistry, a selective contact miticide, insecticide or biocide that controls pests in apple, grapes, pear, pistachio, stone fruits, and the tree nuts group | Sediments |
| Bioallethrin (CAS 260359-57-7) | Agrochemistry, pesticide or acaricide to kill flies and mosquitoes; the d-trans isomer is a more potent insecticide used to control animal parasites | Eggs |
| Pyracarbolid (CAS 24691-76-7) | Agrochemistry, an obsolete anilide fungicide and bactericide that was used for seed treatment and to control a range of fungal diseases including rusts. | Eggs |
| Terbucarb (CAS 1918-11-2) | Agrochemistry, as an insecticide or pesticide in homes, gardens, and agricultural applications | Eggs |
| Resmethrin (CAS 10453-86-8) | Agrochemistry, pyrethroid insecticide registered for control of insects in residential, commercial and industrial settings, and in animal living areas, and to control adult mosquitoes in the interest of public health. | Eggs |
| 3-Hydroxybiphenyl (CAS 580-51-8) | Agrochemistry, as a post-harvest fungicide for citrus fruits and as an environmental food contaminant and antifungal. | Sediments, eggs |
| 1,2,4-trichloro-benzene (CAS 120-82-1) | - Agrochemistry, as a dye carrier, an herbicide intermediate, and as an insecticide against termites  - Industrial chemistry, a heat-transfer medium, a dielectric fluid in transformers, a degreaser, a lubricant, in synthetic transformer oils, and as a solvent in chemical manufacturing | Sediments, eggs |
| 1,3-dichloro-benzene, (CAS 541-73-1)  +  1,2-dichloro-benzene (CAS 95-50-1) | - Agrochemistry, as a fumigant, insecticide, and chemical intermediate, in the production of various herbicides and insecticides  - Industrial chemistry, in the production of pharmaceuticals and dyes | Sediments, eggs |
| Dibutyl phthalate (CAS 84-74-2) | Industrial chemistry, to help make plastics soft and flexible. It is used in shower curtains, raincoats, food wraps, bowls, car interiors, vinyl fabrics, floor tiles, and other products | Sediments, eggs |
| Diethyl phthalate (CAS 84-66-2) | Industrial chemistry, to make plastics more flexible, products in which it is found include toothbrushes, automobile parts, tools, toys, and food packaging, can be released easily from these products, as it is not part of the chain of chemicals (polymers) that makes up the plastic in cosmetics, insecticides, and aspirin | Sediments, eggs |
| 2-methyl-naphthalene (CAS 91-57-6) | Industrial chemistry, produced from coal tar and used to make dyes and resins, to make vitamin K; present in asphalt and smoke from burning cigarettes and wood | Sediments, eggs |

**Fig. S1** Gas chromatography – high-resolution mass spectrometry (GC-HRMS) chromatogram of the total ion current (TIC) of a standard solution at 500 ng  mL^- 1^ for all the target analytes.
